# Sensitivity analysis of the Aquacrop and SAFYE crop models for the assessment of water limited winter wheat yield in regional scale applications

**DOI:** 10.1371/journal.pone.0187485

**Published:** 2017-11-06

**Authors:** Paolo Cosmo Silvestro, Stefano Pignatti, Hao Yang, Guijun Yang, Simone Pascucci, Fabio Castaldi, Raffaele Casa

**Affiliations:** 1 Department of Agriculture Forestry and Nature (DAFNE), University of Tuscia, Viterbo, Italy; 2 Institute of Methodologies for Environmental Analysis (IMAA), Consiglio Nazionale delle Ricerche (CNR), Tito Scalo, Potenza, Italy; 3 National Engineering Research Center for Information Technology in Agriculture (NERCITA), Beijing, China; University of Siena, ITALY

## Abstract

Process-based models can be usefully employed for the assessment of field and regional-scale impact of drought on crop yields. However, in many instances, especially when they are used at the regional scale, it is necessary to identify the parameters and input variables that most influence the outputs and to assess how their influence varies when climatic and environmental conditions change. In this work, two different crop models, able to represent yield response to water, Aquacrop and SAFYE, were compared, with the aim to quantify their complexity and plasticity through Global Sensitivity Analysis (GSA), using Morris and EFAST (Extended Fourier Amplitude Sensitivity Test) techniques, for moderate to strong water limited climate scenarios. Although the rankings of the sensitivity indices was influenced by the scenarios used, the correlation among the rankings, higher for SAFYE than for Aquacrop, assessed by the top-down correlation coefficient (TDCC), revealed clear patterns. Parameters and input variables related to phenology and to water stress physiological processes were found to be the most influential for Aquacrop. For SAFYE, it was found that the water stress could be inferred indirectly from the processes regulating leaf growth, described in the original SAFY model. SAFYE has a lower complexity and plasticity than Aquacrop, making it more suitable to less data demanding regional scale applications, in case the only objective is the assessment of crop yield and no detailed information is sought on the mechanisms of the stress factors affecting its limitations.

## Introduction

Drought is one of the phenomena which most influences agricultural production worldwide, causing significant and occasionally dramatic harvest losses [1]. As recently reviewed by [2], considerable efforts have been made to analyze the complex phenomenon of drought and assess its severity and impact [3–5]. Available approaches, in this context, include the prediction of yield losses in the presence of water shortages, both at the field and at the regional scale, e.g. using remote sensing data [6] coupled to crop models [7]. First requirement herewith, is a crop model suitable to study the impact of drought, i.e. one that can correctly simulate physiological processes related to the soil water status, such as crop transpiration and yield responses to water. Many models are available for this purpose, with varying degree of complexity and predictive performance, such as WOFOST [5, 8], the CERES DSSAT models [9], STICS [10] or CROPSYST [11], among the most widely used. Although these process-based crop models were originally conceived for field scale applications, they are increasingly employed for spatialized regional scale studies [12–13] and remote sensing data assimilation [13–16]. Crop models are usually quite demanding in terms of data requirements and, when applied to large areas, they can be affected by many sources of uncertainty, due to the poor quality of input data (e.g. weather, soil), to the lack of information on management (e.g., sowing dates, fertilization practices, cultivars grown), which can be also variable in space and time, as well as to the model structure [14, 17], to the experience of the model users [18] and to the uncertainty in the data used for their calibration [19–20].

To address these issues, it is possible to follow two different strategies: 1) use a simple model with a reduced number of parameters and inputs, or 2) fix the values of the parameters and input variables which are less influential, in order to reduce as much as possible the number of factors to vary. In all circumstances, however, a preliminary identification of the factors that most affect the targeted outputs of the model is required.

The use of a simple model, with a reduced number of parameters, was proposed, for example, by Duchemin et al. [21], who developed the Simple Algorithm For Yield (SAFY), in order to investigate the perspectives offered by coupling a simple vegetation growth model and ground-based remotely-sensed data for the monitoring of wheat production. The model simulates dynamically leaf area index (LAI) and dry above-ground biomass and crop yield. Being quite simple, it is very attractive for operational applications at a regional scale. It was employed by Chahbi et al. [22] to estimate the dynamics and yields of cereals in semi-arid, low productivity regions in North Africa, confirming the ability of the model for yield prediction. However, the model does not take into account the effects of water or nutrient limitations on plant growth and it does not simulate physiological processes related to the soil water status, such as crop transpiration and yield responses to water. The impact of water deficit is expected to be accounted for by the variation of the effective light-use efficiency, with the idea that this parameter can be calibrated from the time course of the LAI observed from remote sensing. [21] highlight the limits of this assumption, since LAI is an indicator of all agro-environmental stresses considered together, with no possibility to scrutinize the underlying causes for yield reductions. For these reasons, Duchemin et al. [23] and Veloso [24] added a water balance and evapotranspiration component to the model, thus renamed SAFYE.

Alternatively, more complex models can be used, such as Aquacrop [25], developed by the Food and Agriculture Organization (FAO) of the United Nations specifically for the purpose of assessing crop response to water and increasingly used by scientists and agronomists [26–30]. Aquacrop is also very interesting in the context of remote sensing data assimilation, because it employs canopy cover (CC) as a key state variable. CC is easier to retrieve from remote sensing than e.g. LAI, which is difficult to estimate at high values, being subject to saturation of the reflectance signal [31]. In a comparison study of different wheat crop models [32], Aquacrop was found to be the most sensitive to water stress and the simplest when compared to other models, with comparable accuracy. Aquacrop was shown to simulate correctly winter wheat yield under non-limiting [33] and water deficit [12, 29–30, 34] conditions, generally providing better results in wet or moderate water stress years than in very dry years [29], thus requiring additional investigations before applying it to severe drought conditions. After appropriate calibration, Aquacrop could achieve root mean square errors (RMSE) between measured and estimated yields, in the order of 0.27 [34] to 1.29 [30] t ha^-1^, which is comparable to other well established models such as CERES-Wheat, for which RMSE values of 0.17 to 1.2 t ha^-1^ were reported [9].

Regardless of the model used, the identification of parameters and input variables that most affect its outputs is a fundamental problem for all the applications whenever large uncertainty on their values is expected, such as at the regional scale, and when assimilation algorithms are employed. In general, this is also important for model calibration studies, since accurate calibration of a minimum number of parameters is a requirement for all crop models.

A suitable technique for such purpose is sensitivity analysis (SA). The aim of a sensitivity analysis is to determine how sensitive the outputs of a crop model are, with respect to the elements of the model which are subject to uncertainty or variability, i.e. typically input variables and parameters [14]. It is possible to distinguish two different strategies: local and global SA [35]. An extensive overview of SA methods was done by Cariboni et al. [36], who show the inadequacy of local methods for crop models, because of the complexity of the latter and the necessity to know the interaction between parameters. For these reasons Global Sensitivity Analysis (GSA) is considered more adequate in this context. GSA methods evaluate the effect that the simultaneous change of several or all the input factors have on the output of the model in wide ranges of variation [35]. The estimate of sensitivity of the model to each parameter is obtained by varying at the same time many or all the input factors, measuring the combined effect on model outputs. The inconvenience of this methods is the high computational cost and, in case of an excessive number of factors, the difficulty of convergence of the algorithm.

Confalonieri et al. [37] performed a comparison of several SA techniques, applying them to the rice crop model WARM and demonstrating the agreement between rankings of crop parameters from the most to the last relevant. They evaluated the accuracy and the efficiency of each SA method, concluding that resource intensive methods might not be needed to identify the most relevant parameters. In facts, the Morris method, the simplest amongst GSA methods assessed, produced results comparable to those obtained by more computationally expensive methods. If the model is described by a limited number of parameters or it is possible to exclude *a priori* some parameters, it becomes convenient to employ a variance-based method. The most frequently used methods are Sobol and EFAST. The efficiency of these methods is similar, in terms of computational time, but EFAST highlights better than Sobol the influence of interactions between parameters on the variance model [38]. In the literature both are used to analyze the sensitivity of a limited number of parameters [14, 38–39]. Confalonieri [37] proposed a combination of Morris and Sobol methods, to exploit the computational advantages of Morris method to detect the non influential parameters and to reduce the number of parameters when using Sobol. Vanuytrecht et al. [40] used the same strategy, applying at first a screening technique using the Morris method and subsequentially EFAST as variance-based method to examine sensitivity of the yield output of the Aquacrop model for different crops and climates. They showed that the sensitivity to important parameters depends strongly on environmental conditions and it was not possible to establish a ranking of parameters valid for each climatic scenario. This signifies that it would be necessary to perform a sensitivity analysis, possibly a simple screening method, for the scenarios in which the model will be used. Here, the plasticity of the model, i.e. the tendency to change its behavior and sensitivity under different conditions, is thus determining the need for an extent of SA. From the operational point of view, output sensitivity and plasticity of a crop model are essential informative characteristics when one wants to assimilate remote sensing data into crop models at the regional scale.

In the present study, the sensitivity of two models, suitable for the assessment, by means of remote sensing data assimilation, of wheat yield in water limited conditions in regional scale studies, was assessed. These models are: Aquacrop [25], including a larger number of parameters and input factors, but usually achieving accurate estimates of wheat yield [27–30], and SAFY, having a reduced number of parameters, but generally providing less accurate yield estimates [21–24].

The objective of the present work is to study the complexity and plasticity of these two models in a comparative manner, more specifically focusing on the global sensitivity of the winter wheat yield to model parameters and input variables, in a wide range of water limited conditions occurring during the wheat growth season. A previous global sensitivity analysis was carried out for Aquacrop by Vanuytrecht et al. [40], but no assessment of its plasticity has been performed. This is a crucial aspect, relevant for all the users of the model in large scale spatialized applications. For SAFY no previous global sensitivity analysis has been reported yet.

## Materials and methods

### Crop models used

#### Aquacrop

Aquacrop is a widely used "water driven" productivity model that simulates canopy cover (CC), biomass and yield of a crop mainly as a function of the water productivity, i.e. the biomass produced per unit of water transpired by the vegetation, normalized for atmospheric evaporative demand and air CO_2_ concentration [25–30; 33–34]. Input variables include weather data, consisting of daily maximum and minimum temperature, evapotranspiration and rainfall, as well as soil properties and agronomic management information, including some related to the crop (e.g. sowing date). In the version of Aquacrop used in the present work (version 4.0) there are 45 parameters (S1 Table), defining the crop physiological and developmental responses to environmental factors and to soil water and salinity stresses. Of all these parameters, 29 are considered as conservative, i.e. crop specific, but not changing with cultivar, time, management practices, geographic location or climate [41]. These parameters are not supposed to require a local calibration for a well studied crop such as wheat, but would need to be calibrated using data from multiple location for a species new to Aquacrop.

Crop development is limited by upper and lower temperature thresholds and is determined by a set of phenological parameters (e.g. *eme*, *flo*, *mat*, *sen*) which specify the length (in days or in growing degree days) of each development phase, e.g. from sowing to emergence (*eme*), flowering (*flo*), maturity (*mat*) or senscence (*sen*).

Canopy cover (*CC*) is the main state variable describing the growth of the foliage. Its dynamics, in non limiting conditions, are regulated by parameters setting the initial canopy cover, its growth rate coefficient (*CGC*), the maximum *CC* potentially achievable (*CCx*) and its decline rate coefficient (*CDC*). Water stress, as well as soil salinity or limited fertility, limits or delays the *CC* development through stress coefficients.

The amount of water transpired by the crop is a function of *CC*. Cumulative biomass production is obtained as the sum of the daily ratio between crop transpiration and ET_0_ for each day of the crop cycle. The proportional factor between biomass and transpiration ratio is the normalized Water Productivity (*pwp*) (Eq 1):

$$B_{n}=pwp\cdot \Sigma_{i=1}^{n}\left(\frac{Tr_{i}}{ET_{0}{}_{i}}\right)$$ (1)

where *B*_*n*_ is the cumulative aboveground biomass production at day n (g m^-2^); *Tr*_*i*_ is the daily crop transpiration (mm day^-1^); ${{ET}_{0}}_{i}$ is the daily reference evapotraspiration (mm day^-1^); *i* is the day of crop cycle (from 1 to n) and *pwp* is the normalized crop water productivity.

The yield is calculated by multiplying the final biomass by a harvest index [41]. The influence of water stress on crop development is represented by a number of stress coefficients, which describe the impact of water stress on canopy development, transpiration and harvest index due to limited water availability. Additionally, temperature limitations on biomass production and pollination are considered. A more detailed description of the Aquacrop model is presented by Raes et al. [41].

#### SAFYE

The original Simple Algorithm For Yield (SAFY) proposed by [21] is based on the Monteith concept [42], which links the production of total dry phytomass to the photosynthetically active portion of solar radiation (PAR) absorbed by the crop. SAFY simulates, on a daily time step, three main state variables from emergence of the crop to the end of its senescence. These are: Dry Above-ground Mass (DAM), Leaf Area Index (LAI) and Grain Yield (GY).

The daily production of dry above-ground phytomass (Δ*DAM*) depends on the incoming global radiation (*Rg*) through three parameters: 1) climatic efficiency (*Pgro_R2P*) which is the ratio of incoming photosynthetically active to global radiation; 2) light-interception efficiency (*Pgro_Kex*), which affects the fraction of photosynthetically active radiation absorbed by the canopy (APAR) and 3) effective light-use efficiency *Pgro_Lue* (g MJ^-1^), which is the ratio of photosynthetically energy produced as DAM from APAR.

Furthermore, a temperature stress function *F*_*T*_(*Ta*) guarantees that the daily rate of biomass production of vegetation (Δ*DAM*) increases as the air temperature is closer to the optimum temperature (*Ptfn_Topt*), and goes to zero out of the range between minimum and maximum critical temperatures. The final equation of Δ*DAM* is:

$$\Delta DAM=R_{g}\cdot Pgro\_R2P\cdot Pgro\_Kex\cdot Pgro\_Lue\times F_{T}\left(Ta\right)=APAR\times Pgro\_Lue\times F_{T}\left(Ta\right)$$ (2)

The light-interception efficiency depends on the green leaf area index (GLAI) and a light interception coefficient *Pgro_Kex* according to Beer's law. The development of GLAI is split into two phases. In the first phase GLAI increases, starting at crop emergence and ending at the beginning of senescence. In the second phase, between the beginning and the end of senescence, GLAI decreases with a rate defined by senescence parameters.

The third state variable simulated by SAFY is the grain yield. It is calculated from DAM by means of a proportionality factor *Pgro_P2G*, corresponding to the harvest index. A detailed description of SAFY was presented by Duchemin et al.[21].

In the present work, a modified version of SAFY, called SAFYE was used, introducing a dependence of biomass yield on crop water stress, adopting the same modification as used by [23] and [24]. These modifications were introduced since they allow an explicit assessment of the crop response to water availability and the characterization of water stress, which was lacking in the original model. The dry above-ground phytomass rate of change (Δ*DAM*) is multiplied by a water stress factor K_s_, a dimensionless transpiration reduction factor dependent on available soil water, ranging between 0 and 1 [43]. K_s_ is calculated from the total available soil water in the root zone (TAW) and readily available soil water in the root zone (RAW), resulting from a simplified water balance driven by crop evapotranspiration, as described by the FAO irrigation and drainage paper n. 56 [43]. Δ*DAM* used in the modified version of SAFY is computed as:

$$\Delta DAM=APAR\cdot Pgro\_Lue\cdot F_{T}\left(Ta\right)K_{s}$$ (3)

The version of SAFYE used in this work has 23 parameters in total, of which 10 were added in the present modified version to the 13 of the original SAFY [21]. Of these 23 parameters, 13 can be considered as conservative, i.e. theoretically not needing a local calibration [22], whereas 9 are cultivar specific and 1 depends on management factors, as listed in S2 Table. Additionally there are 8 input variables related to soil properties and management factors and 7 daily weather variables required: solar radiation, minimum and maximum air temperature, minimum and maximum relative humidity, mean wind speed and reference evapotranspiration.

SAFYE was tested with field data collected on winter wheat in Xiaotangshan (40.17°N, 116.43°E), near Beijing (China) during four years (2008–2011), within an experimental trial in which different sowing dates were used, according to a randomized block design with three replicates. In the first three years, three sowing dates were adopted: 28 September, 7 and 20 October in 2008–2009; 25 September, 5 and 15 October in 2009–2010; 25 September, 5 and 15 October in 2010–2011. The last year (2011–2012) only one sowing date was used: 25 September. The area of each plot was 100 m^2^ in 2008–2010 and 300 m^2^ in 2011. Weed control, pest management and fertilizer application were performed according to the local standard practices. The biomass and the leaf area index (LAI) were sampled 5–6 times during each growing season. The LAI-2000 Plant Canopy Analyzer (LI-COR Inc., Lincoln, NE, USA) was used for the determination of LAI. Aboveground biomass was collected using random sampling of a 0.25-m^2^ area, with four replicates per plot. All samples were oven dried at 70°C to a constant weight. The grain yields of each plot with three replicates for each treatment were obtained by randomly sampling a 1.5 m^2^ area. Further details on this experimental dataset are provided by [30].

The performance of the model, previously validated by [23] and [24], was assessed by comparing simulated and measured data by computing the Root Mean Squared Error (RMSE) and the relative RMSE, i.e. RRMSE the ratio between RMSE and the mean of the measured data.

### Sensitivity analysis strategy

Following a widely used procedure [37, 40], the SA strategy used in this study was to apply preliminarily a screening method in order to be able to exclude non-influential parameters of the models. In the second step, a variance based method is applied, which requires a longer computational time, but allows the assessment of interactions and higher order effects.

The screening method of Morris [44], followed by the variance based method Extended Fourier Amplitude Sensitivity Test (EFAST) [35] were used in this work The latter method complements the first, which does not allow to quantify non linear and second order effects. Besides, although considered a GSA method, the experimental part of the Morris method is composed of individually randomized one at a time (OAT) experiments and sensitivity measures are typically considered qualitative (i.e., ranking significant input factors), but not necessarily quantitative in regard to the degree of significance [45].

#### Morris method

The Morris Method [44] is designed for the quantification of elementary effects that the variation of input factors (parameters and input variables) produce on model outputs. The method determines whether the effects are negligible, linear and additive, non-linear or involving interactions with other factors [46].

The experimental design is structured in groups of points, called trajectories. In this study the number of points was chosen as equal to the number of factors, i.e. model input variables or parameters, so that k = p. In this case the number of trajectories (r) has been set to 20, as a compromise between the minimum value suggested by Campolongo et al. [46], i.e. 10 and the value used by [40], i.e. 25, in order to have limited computational cost. Morris [44] suggested to use the mean (μ) and the standard deviation (σ) of the finite distribution of elementary effects associated with the i-th input parameter as sensitivity indices. Empirical studies [46] have shown that the absolute value of mean (μ*) can be considered as a “total sensitivity index”. It is sufficient that μ* is below a threshold value to consider a parameter as negligible. In this study, a parameter or input variable was considered as non influential when, in all the scenario considered, μ* was below a threshold value of 0.1 t ha^-1^ of grain yield, which was the only output variable examined in this study. This threshold value was chosen arbitrarily, assuming to represent a yield error well below what has been reported to be the typical range of error found when employing the models [23–24, 29–30, 33–34].

#### Extended Fourier Amplitude Sensitivity Test (EFAST)

EFAST is an evolution of the Fourier Amplitude Sensitivity Test (FAST), a SA method based on the decomposition of the Sobol variance [35]. It calculates sensitivities indices using the total variance of the output of the model and the conditional variances depending on the parameters. The interaction among factors can be quantified by calculating a main sensitivity index (S_i_) and an index of total sensitivity (ST_i_), i.e. the sum of the main effect plus the interaction between the variation of parameters terms to all orders. S_i_ and ST_i_ range between 0 and 1, with higher values indicating more important effects.

In the present study, EFAST has been used subsequently to the application of the Morris method, analyzing only the parameters found to be non-negligible. Since there is not an objective way of establishing a threshold value for considering a parameters as negligible or influential, we decided to adopt a threshold of ST_i_ of 0.1 (i.e. 10% of the output variance), on the basis of an analysis of the literature, since this was the most frequently used value [17, 36–37, 45].

For this study a Matlab (Mathworks Inc., MA, USA) implementation of the EFAST and Morris algorithms was used [47].

### Range of variation of model parameters

The range within which the parameters and input variables were allowed to vary during the GSA was defined in order to represent the uncertainty on their values in the context of a regional scale model application. For both Aquacrop and SAFYE, the parameters considered as conservative, i.e. not suppose to be varied for a given crop species, were allowed to vary within a maximum range of ±33% of a mean nominal value obtained from the literature as detailed in S1 and S2 Tables. In facts, the calibration results of different studies on wheat [29–30, 33–34] suggest that even for these parameters a limited range of variation is needed. Thus we adopted for these parameters a range resulting from the values used these studies, including them in the SA, as also done by Vanuytrecht et al. [40]. The range of variation of the other parameters and input variables was also defined, as specified in S1 and S2 Tables, according to the literature [48–49] or expert knowledge on the variability of each factor likely to be faced when applying the models in a regional scenario. It should be noted that this application context is rather different from the typical field scale model application, in which knowledge of many soil input variables (e.g. soil texture) or management factors (e.g. sowing date) is generally available. The GSA for Aquacrop was carried out for 9 input variables and 45 parameters, of which 29 were considered as conservative, as presented in the S1 Table.

For SAFYE, the range of variation of the parameters and input factor was set using the same criteria used for Aquacrop, adopting whenever possible the same ranges for equivalent parameters in the two models, as detailed in S2 Table. The GSA was carried out for 8 input variables and 23 parameters, of which 13 were considered as conservative.

### Assessment of model complexity and plasticity

The two models, Aquacrop and SAFYE, were compared in terms of two indicators derived from the GSA results: complexity and plasticity. Complexity is the parsimony of the model in representing the biophysical system, providing information about the amount and relevance of model parameters and inputs [50]. Plasticity is defined as the aptitude of a model to change the sensitivity to its parameters and inputs when the conditions of application change [51].

In this study we choose to represent the complexity through the parameter ratio (R_p_) introduced by Confalonieri et al. [52], i.e. the proportion of relevant (sensitive) factors over the total number of factors tested in the SA. All the parameters and input variables for which the ST_i_ was higher than 0.1 were considered as relevant in this study.

According to Confalonieri et al. [52], plasticity can be quantified using an index L:

$$L=TDCC\cdot e^{\sigma SAM^{-1}}$$ (4)

where TDCC is the top-down concordance coefficient presented by Iman and Conover [53] and σ_SAM_ is the standard deviation of a normalized agrometeorological indicator (SAM) proposed by Confalonieri et al [50]. TDCC, ranging between 0 and 1, is considered suitable for comparing parameters rankings obtained from SA carried out under different conditions. It has the capability of emphasizing the agreement among rankings assigned to relevant parameters and of deemphasizing the disagreement among those of less important parameters [50].

The normalized synthetic agrometeorological indicator SAM [52], ranging between -1 (corresponding to maximum drought) and +1, is computed as:

$$SAM=\frac{Rain-{ET}_{0}}{Rain+{ET}_{0}}$$ (5)

where *Rain* (mm) and *ET*_*0*_ (mm) represent respectively the cumulative rainfall and reference evapotraspiration. Confaloneri et al. [52] showed that SAM is a very useful standardized indicator for characterizing the conditions of applications of crop models. The plasticity index L proposed by Confalonieri et al. [51] ranges from 0 to about 1.51, with the highest plasticity at 0. This indicator couples model response, represented by TDCC, with the variability of environmental conditions, represented by the standard deviation of SAM.

### Climatic scenarios

The meteorological data used in this study to drive the model simulations for the sensitivity analysis were obtained from three sites representing contrasting environments, in terms of temperature extremes and water availability during the winter wheat growth season (Table 1). They were selected in order to encompass a large variability of water limitation patterns during the winter wheat growth season, either in the early phase (autumn to beginning of winter) or in the full wheat development phase (end of winter to spring).

**Table 1:** Summary of the climatic data sets used in this study. Cumulative rainfall and reference evapotranspiration (ETo), average maximum and minimum temperature, evapotranspiration deficit (rain-ETo) and synthetic agrometeorological indicator (SAM) [52], in the most crucial periods of wheat growth, for the years used in the sensitivity analysis study and for the long term climatic data (30 years).

Autumn-Winter (Oct-Dec)									Winter-Spring (Feb-Jun)							
Site	Year	Rain (mm)	ETo (mm)	Tmin (°C)	Tmax (°C)	Deficit ET (mm)	SAM		Year	Rain (mm)	ETo (mm)	Tmin (°C)	Tmax (°C)	Deficit ET (mm)	SAM	
Yangling	1994	236.6	392.7	10.3	18.4	-156.1	-0.2	Wet	1995	189.5	817.1	9.9	22.0	-627.6	-0.6	Dry
	1986	197.1	405.5	8.8	18.0	-208.4	-0.3	Dry	1987	442.3	726.6	8.7	19.9	-284.3	-0.2	Wet
	long term mean	266.0	391.7	9.3	18.3	-125.8	-0.2		long term mean	306.6	761.9	9.1	20.3	-455.4	-0.4	
Viterbo	2002	210.4	138.3	9.2	18.3	72.1	0.2	Dry	2003	106.2	538.1	8.1	22.7	-431.9	-0.7	Dry
	2009	350.6	113.1	6.3	15.9	237.5	0.5	Wet	2010	508.2	422.1	7.2	18.3	86.1	0.1	Wet
	long term mean	270.8	129.4	6.5	15.6	141.3	0.4		long term mean	276.9	460.8	7.1	18.8	-183.9	-0.2	
Xiaotangshan	2010	139.8	122.8	5.3	15.0	17.0	0.1	Wet	2011	432.5	508.5	8.2	18.9	-76.0	-0.1	Wet
	2011	116.8	250.2	5.9	14.9	-133.4	-0.4	Dry	2012	481.9	785.1	8.3	18.4	-303.2	-0.2	Dry
	long term mean	37.59	128.7	0.5	10.9	-91.1	-0.5		long term mean	152.7	491.2	7.1	18.9	-338.5	-0.5	

The Xiaotanshan site (lat. 40.17°N, long. 116.43°E, alt. 57 m), near Beijing (China), is characterized by a continental climate, with a cold dry winter and a hot wet summer, belonging to the Koppen-Geiger [54] class Dwa (cold, dry winter, hot summer), i.e. monsoon-influenced, hot-summer humid continental climate.

The Yangling site (lat. 34.27°N, lon. 108.09°E, alt. 460 m), in the Chinese Province of Shaanxi, is located within a Monsoon-influenced humid subtropical area, falling within the Cwa Koppen-Geiger class (temperate, dry winter, hot summer), characterized by high evapotranspirative deficit in spring.

Viterbo (lat. 42.72°N, long. 12.12°E, alt. 310 m), Central Italy, has a typical Mediterranean climate with wet autumn and dry hot summer, falling into the Koppen-Geiger class Csa (temperate, dry summer, hot summer).

The dataset consisted of daily rainfall, temperatures (mean, maxima and minima), relative humidity and wind speed. Solar radiation was calculated from the temperature range using the relationship proposed by Bristow and Campbell [55]. Reference evapotraspiration was calculated using the FAO Penman-Monteith method [43], using all the available variables (temperature, humidity, solar radiation and wind speed).

To quantify the degree of crop water stress in the three sites, we calculated the evapotranspirative deficit, i.e. the difference between rainfall and reference evapotranspiration (ETo) and the normalized synthetic agrometeorological indicator (SAM) [52]. This index allows an easy comparison among sites and years, though it is only a partial indicator of the seasonal conditions impacting crop growth, since it does not take into account temperature extremes.

**Fig 1:**
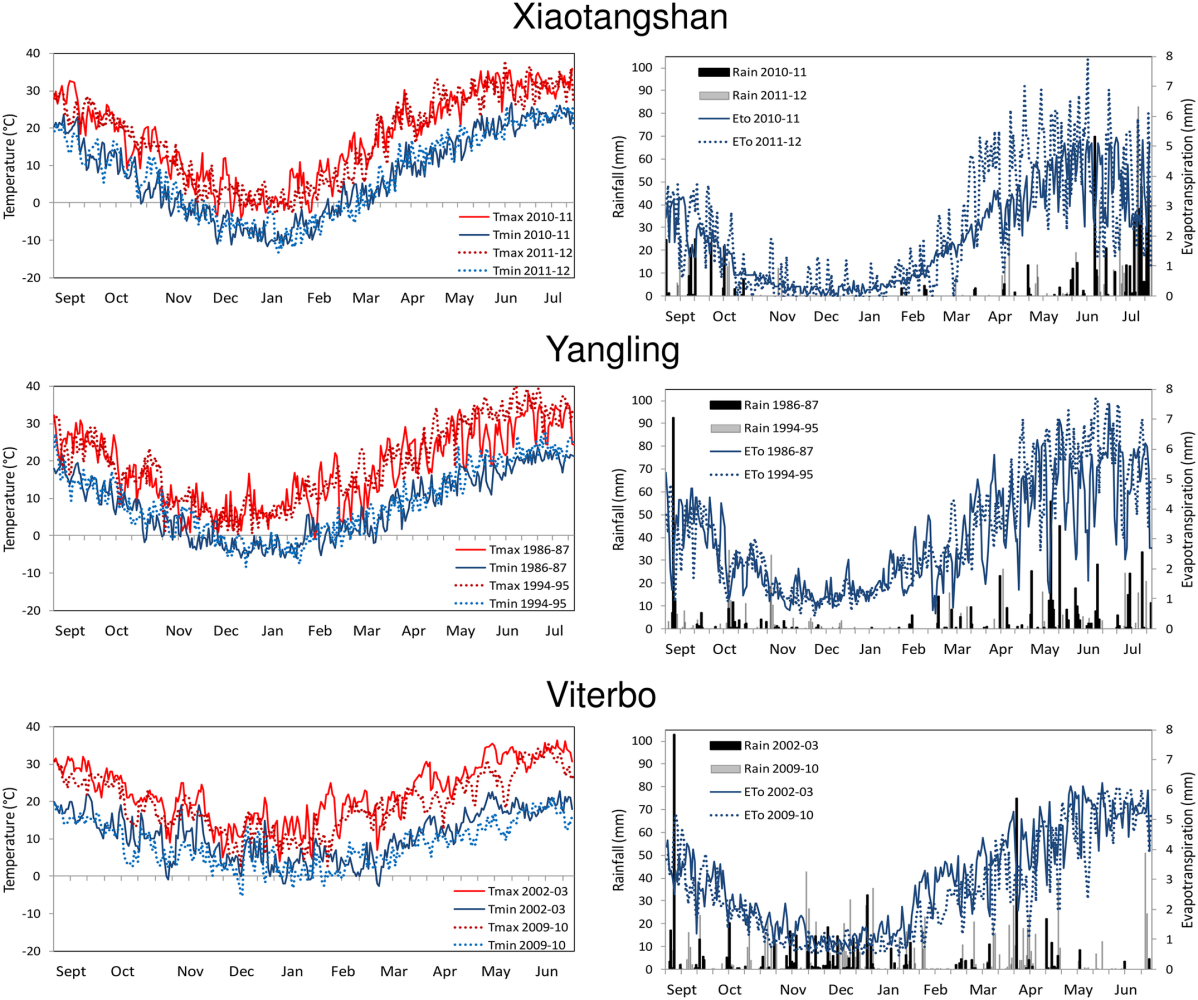
Meteorological datasets used in the sensitivity analysis study. Time trends of daily maximum and minimum air temperature (left), rainfall and reference evapotranspiration (right) for the sites of Xiaotanshan (top), Yangling (centre) and Viterbo (bottom), for the two years considered in each study site.

Based on such indicators, we selected, for each site, two winter wheat growth seasons (Fig 1), spanning from the 1st of September to 31st of July, characterized respectively by relatively dry or wet conditions. These were selected on the basis of the evapotranspiration deficit and SAM occurring in the periods considered as most important for winter wheat growth (Table 1), i.e. from October to December (autumn to beginning of winter) and from February to June (end of winter to spring).

## Results

### Assessment of the SAFYE model

**Fig 2:**
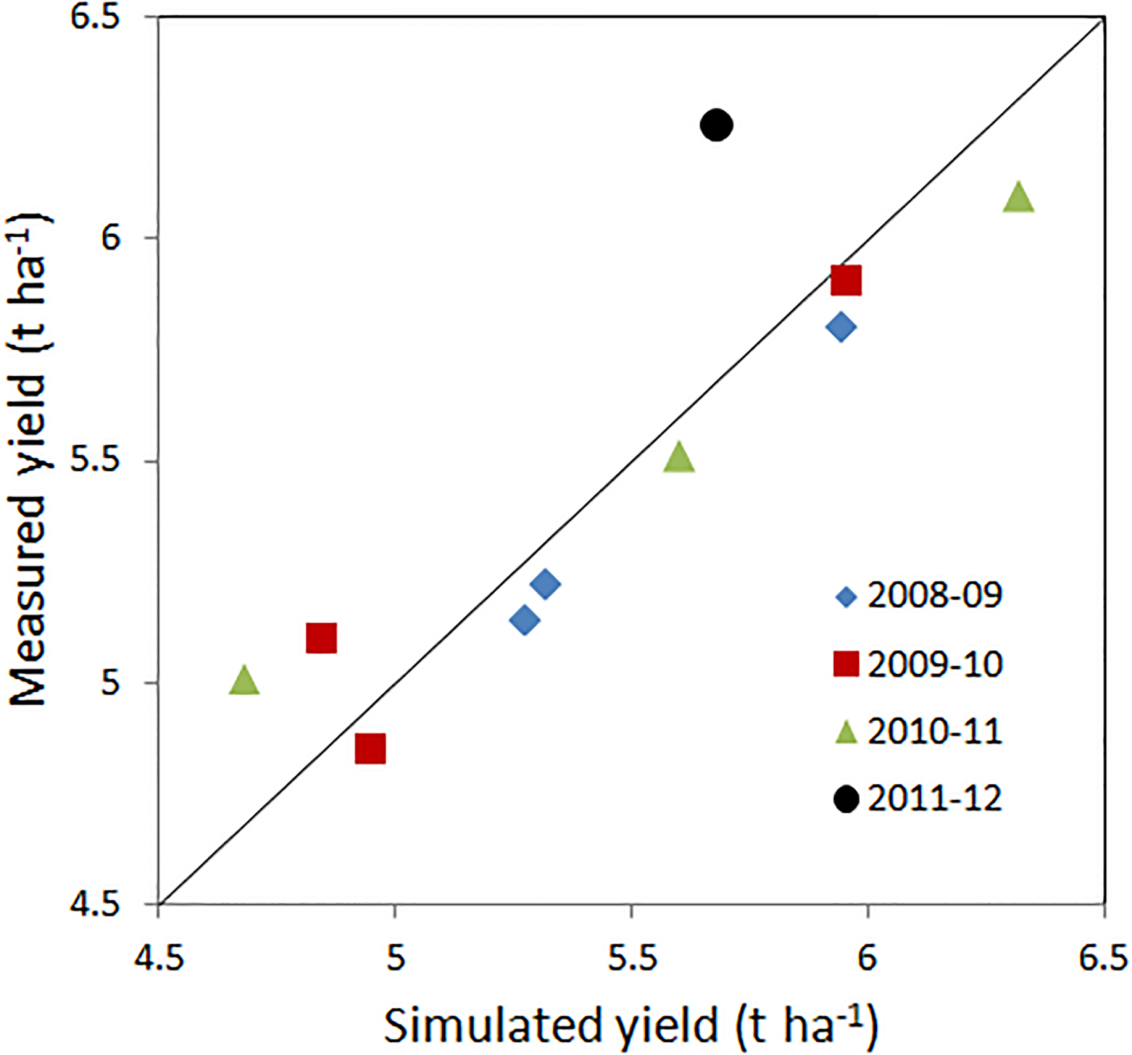
Relationship between measured and simulated grain yield in winter wheat with the SAFYE model, across 4 years at Xiaotanghan.

The version of the SAFYE model used in the present work was able to simulate quite accurately the winter wheat grain yield of the Xiaotangshan dataset (Fig 2), with an overall RMSE of 0.25 t ha^-1^ and a RRMSE of 5% for the final grain yield estimation across all years and sowing dates. The time trends of simulated above ground biomass and LAI were in agreement with measured data for most of the sowing dates, although the time of simulated grain maturity was slightly delayed as compared to the actual harvest dates (S1 Fig).

SAFYE lead to a small improvement in the estimation of grain yield as compared to the original version of the SAFY model [21] for which the RMSE was 0.27 t ha^-1^, for this dataset.

### Application of Morris methods

**Fig 3:**
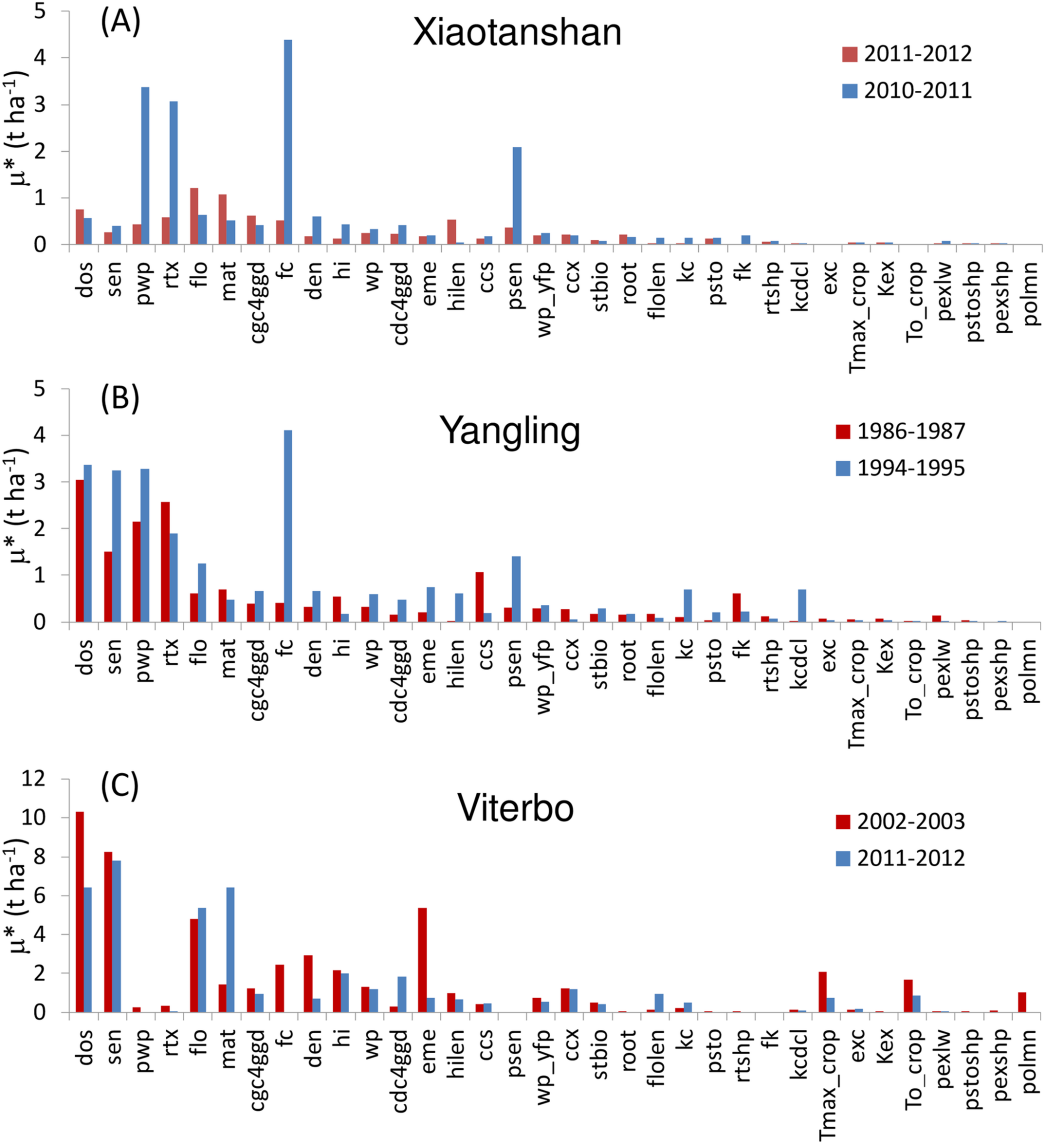
Values of Morris mean effect (μ*) for the Aquacrop model using climatic scenarios for Xiaotanshan (a), Yangling (b) and Viterbo (c) for two crop growth seasons each. Bars in red identify drier years, bars in blue wetter years. Parameters abbreviations are given in S1 Table. Parameters with mean μ*values lower than 0.1 t ha^-1^ have been omitted.

**Fig 4:**
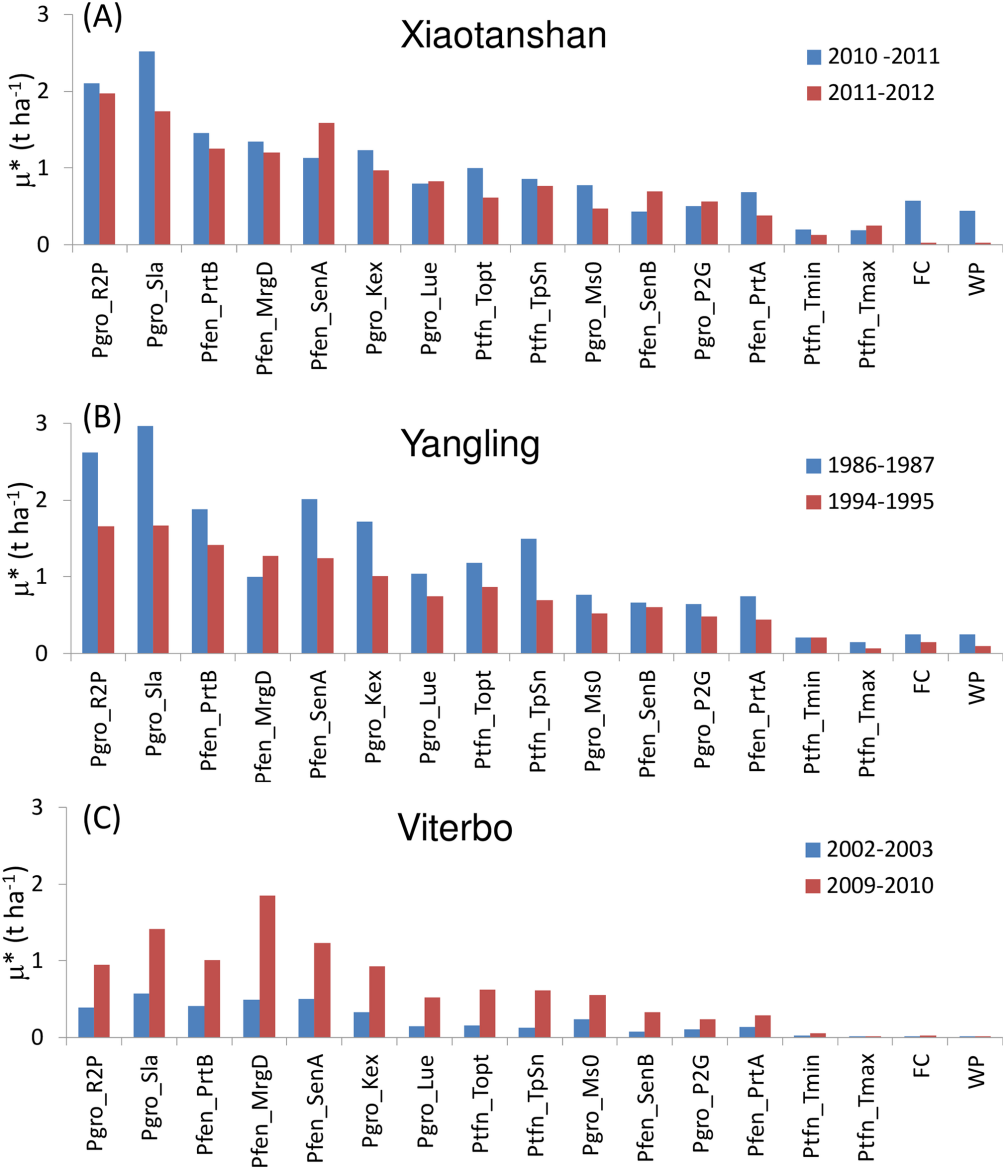
Values of Morris mean effect (μ*) for the SAFYE model using climatic scenarios for Xiaotanshan (a), Yangling (b) and Viterbo (c) for two crop growth seasons each. Bars in red identify drier years, bars in blue wetter years. Parameters abbreviations are given in S2 Table. Parameters with μ*values lower than 0.1 t ha^-1^ have been omitted.

The results of the Morris method (both for Aquacrop and SAFYE) showed that the value of μ* was strongly influenced by the climatic scenarios employed. In facts, values of μ* were rather different not only between the sites, but they also varied significantly among the years analyzed (Figs 3 and 4). However, there was a number of factors that were consistently non-influential, i.e. with μ* values lower than 0.1 t ha^-1^, that could be identified for both models, which was the main objective of the application of this screening method.

In general, higher values of μ* were observed for Aquacrop than for SAFYE. In particular, very high values of μ* were found for Aquacrop in Viterbo, for which the highest sensitivity was for the day of sowing (*dos*), and for the temperature sum (GDD) until sensecence (*sen*), grain physiological maturity (*mat*) and until flowering (*flo*). The latter two parameters seemed especially influential in the wetter year. Also for Yangling these parameters resulted quite important and the influence of *flo* and *sen* was similarly higher in the wetter year, whereas they resulted less influential but still not negligible in Xiaotangshan. For the Xiaotangshan site though, the sensitivity of yield to these parameters was still noticeable, but less strong than that of other factors such as soil field capacity (*fc*) and wilting point (*pwp*). The latter two input variables were also very influential for Yiangling and in both sites their μ* values were higher in the relatively wetter years. It should be noted that these two sites had a remarkable evapotranspirative deficit also in the relatively wetter years (Table 1) and it could be expected that in these conditions the influence of soil water holding characteristics, regulating rain water storage in the root zone, is more important when there is sufficient rain during the growth season, than when rainfall is reduced. A similar pattern, i.e. stronger influence in wetter years in these two sites, appeared also for two other parameters linked to soil water stress: the soil water depletion factor for canopy senescence (*psen*) and for stomatal control (*psto*).

Overall, the SA for Aquacrop shows that all the factors categorized by [40], as related to the development of green canopy, as well as flowering, can be considered as influential. These include both phenological parameters, such as the temperature sum to emergence (*eme*), to crop maturity (*mat*) and the length of flowering (*flolen*), in addition to *sen* and *flo* already mentioned, as well as parameters describing the increase (*cgc4gdd*) or decline (*cdc4gdd*) of the canopy cover. Three parameters related to root development resulted non negligible (i.e. with μ* higher than 0.1 t ha^-1^): most notably maximum rooting depth (*rtx*) and the growing degree days (GDD) to reach maximum root depth (*root*), especially for the Xiaotangshan and Yangling sites. All the parameters related to air temperature stress were non-negligible, with the base temperature below which crop development stops (*To_crop*) and below which pollination starts to fail (*polmn*), especially influential in Viterbo in 2002–03 where low temperatures occurred in March (Fig 1). It is interesting to note that *To_crop* was not influential in Xiaotangshan in which temperatures were well below the range used for this parameter, whereas in Viterbo the temperatures recorded in winter tended to be close to the range used for *To_crop*. Also parameters linked to crop transpiration (*kc* and *kcdcl*), to crop water productivity (*wp* and *wp_yfp*) and to the harvest index (*hi* and *hilen*) were found to be influential. The climate scenarios (sites and years) influenced the rankings of the parameters on the basis of the μ*, as measured by the top-down concordance correlation (TDCC) which overall had a value of 0.47 (in a range 0 to 1). The correlation between the results of the sensitivity analysis of the different scenarios was slightly lower among the wet years (TDCC = 0.49) than among the dry years (TDCC = 0.51), highlighting more differences among the previous. The correlation within sites was higher for Xiaotangshan and Yangling, TDCC of 0.7 and 0.75, than for Viterbo which had a correlation of 0.63 among the sensitivity analysis results of the dry and wet year.

Despite the differences of climate and sites, it is possible to distinguish a group of factors which was constantly negligible, i.e. for which μ* was always less than the 0.1 t ha^-1^ threshold (Table 2).

**Table 2:** List of Aquacrop factors that were found to have a negligible influence on grain yield, i.e. with Morris total sensitivity index μ* <0.1 t ha^-1^, in all the climatic scenarios of the present study.

Name of parameter or input variable	Type	Process affected (from [41])	Description
*polmx*	conservative parameter	Air temperature stress	Maximum air temperature above which pollination starts to fail
*evardc*	conservative parameter	Crop transpiration	Effect of canopy cover in reducing soil evaporation in late season stage
*cpco2*	conservative parameter	Crop water productivity	Crop performance under elevated atmospheric CO_2_ concentration
*rtmin*	management input	Development of root zone	Minimum effective rooting depth
*rtexup*	cultivar specific parameter	Development of root zone	Maximum root water extraction in top quarter of root zone
*rtxlw*	cultivar specific parameter	Development of root zone	Maximum root water extraction in bottom quarter of root zone
*exc*	conservative parameter	Harvest Index	Excess of potential fruits
*hipsveg*	conservative parameter	Harvest Index	Coefficient describing positive impact on HI (harvest index) of restricted vegetative growth during yield formation
*hingsto*	conservative parameter	Harvest Index	Coefficient describing negative impact on HI of stomatal closure during yield formation
*hipsflo*	conservative parameter	Harvest Index	Possible increase of HI due to water stress before flowering
*hinc*	conservative parameter	Harvest Index	Allowable maximum increase of specified HI
*Ssf*	management input	Soil fertility stress	Soil fertility/salinity stress coefficient
*ecss*	conservative parameter	Soil salinity stress	Electrical Conductivity of soil saturation extract at which crop can no longer grow
*ecsss*	conservative parameter	Soil salinity stress	Electrical Conductivity of soil saturation extract at which crop starts to be affected by soil salinity
*fk*	management / environment	Soil water stress	Evaporation decline factor for stage II
*Ksat*	management input	Soil water stress	Saturated hydraulic conductivity
*psenshp*	conservative parameter	Soil water stress	Shape factor for water stress coefficient inducing early senescence
*ppol*	conservative parameter	Soil water stress	Soil water depletion factor for pollination: upper threshold (fraction TAW)
*rew*	management input	Soil water stress	Readily evaporable water from top layer
*pexup*	conservative parameter	Soil water stress	Soil water depletion factor for canopy expansion: upper threshold, fraction of total available water (TAW)
*anaer*	cultivar specific / environment	Soil water stress	Anaerobic point below saturation limiting aeration
*cn*	management input	Soil water stress	Soil Curve Number

These include all the factors linked to soil salinity or fertility stress, as well as some parameters related to the harvest index (*exc*, *hipsveg*, *hingsto*, *hipsflo* and *hinc*) and *cpco2*, i.e. crop performance under elevated atmospheric CO_2_, which was obviously irrelevant since CO_2_ was kept constant in the simulations. Some soil water stress factors were negligible, though others of this category were influential as discussed above.

The SA for SAFYE highlighted a set of factors with μ* values higher than the threshold of 0.1 t ha^-1^ established for considering them as influential. This set was approximately the same in all experimental sites, although the values of μ* were generally higher in Yangling and in Xiaotanshan, as compared to Viterbo, for which the highest values were found in the driest year. This was in contrast with the other two sites, for which higher μ* values were generally found in the wetter years, especially in Yangling.

Similarly to what was found for Aquacrop, higher values of μ* for field capacity (*FC*) and wilting point (*WP*) were found in the wetter years in Xiatangshan and Yangling, though in this case they were comparatively less influential than other parameters. The emergence date (*Pfen_MrgD*), which can be considered equivalent to the sowing date in Acquacrop, since that input is not used in SAFYE, was the most influential factor for the dry year in Viterbo, similarly to what found for Aquacrop. In that year the drought and high temperatures occurring in the late spring caused an earlier senesce and termination of the growth cycle, thus sowing date would have been crucial, allowing a longer or shorter time period for photosynthetic assimilation. Specific leaf area (*Pgro_Sla*), used in the model to convert biomass into leaf area, was found to be very influential in all sites. The same was true for the other parameters related to green canopy development, such as the two parameters appearing in the function regulating the partitioning of biomass to leaves (*Pfen_PrtA* and *Pfen_PrtB*), as well as parameters regulating the senescence (Pfen_SenA and *Pfen_SenB*). The conversion factor of solar radiation into PAR (*Pgro_R2P*) had a very strong influence on grain yield, especially at Xiaotangshan and Yangling where more variability in irradiance levels were found than at Viterbo (data not shown, but it can be inferred from ETo patterns in Fig 1). All the parameters related to air temperature stress were also influential (*Ptfn_Topt*, *Ptfn_TpSn*, *Ptfn_Tmin*, *Ptfn_Tmax*). As expected the two key parameters affecting PAR absorption by the canopy (*Pgro_Kex*) and its conversion into biomass (*Pgro_Lue*) were very important, similarly to the parameter regulating the partitioning of biomass to grain (*Pgro_P2G*).

The factors that were found to have a negligible impact, i.e. with μ* values lower than 0.1, were all those introduced in SAFYE (with the exception of soil field capacity and wilting point), i.e. factors linked to transpiration, root development and water stress (Table 3).

**Table 3:** List of SAFYE factors that were found to have a negligible influence on grain yield, i.e. with Morris total sensitivity index μ* <0.1 t ha^-1^, in all the climatic scenarios of the present study.

Name of parameter or input variable	Type	Process affected	Description
*Kc_tab_ini*	Conservative parameter	Crop transpiration	Crop transpiration coefficient at the initial development stage
*Kc_tab_mid*	Conservative parameter	Crop transpiration	Crop coefficient at mid stage
*h_max*	Non conservative parameter	Crop transpiration	Maximum crop height
*Kc_tab_end*	Conservative parameter	Crop transpiration	Crop coefficient at final stage
*SMT_sen*	Non conservative parameter	Development of green canopy cover	Temperature sum to complete senescence
*Pfen_stel*	Non conservative parameter	Development of green canopy cover	Temperature sum at 10% canopy cover, i.e. stem elongation
*Pfen_flw*	Non conservative parameter	Flowering	Temperature sum to flowering
*Rd_tab_end*	Input variable	Root development	Root depth at end of growth
*Rd_tab_ini*	Input variable	Root development	Root depth at emergence
*p_tab_end*	Conservative parameter	Soil water stress	Fraction of readily available water at the final stage
*p_tab_mid*	Conservative parameter	Soil water stress	Fraction of readily available water at the mid stage
*De*	Input variable	Soil water stress	initial cumulative depth of evaporation
*p_tab_ini*	Conservative parameter	Soil water stress	readily available water as fraction of totally available water at the initial stage
*Dr*	Input variable	Soil water stress	initial root zone depletion

The correlation among the results obtained in the different scenarios was much higher with SAFYE than for Aquacrop, with an overall TDCC of 0.92. Although values of TDCC were generally much higher, similarly to what was found for Aquacrop the results of the wet years had a lower correlation (TDCC = 0.9) than those of dry years (TDCC = 0.95). Again, the dry and wet scenarios at Viterbo (TDCC = 0.90) were more different than at Xiaotangshan (TDCC = 0.93) and Yangling (TDCC = 0.98).

### Application of EFAST method

**Fig 5:**
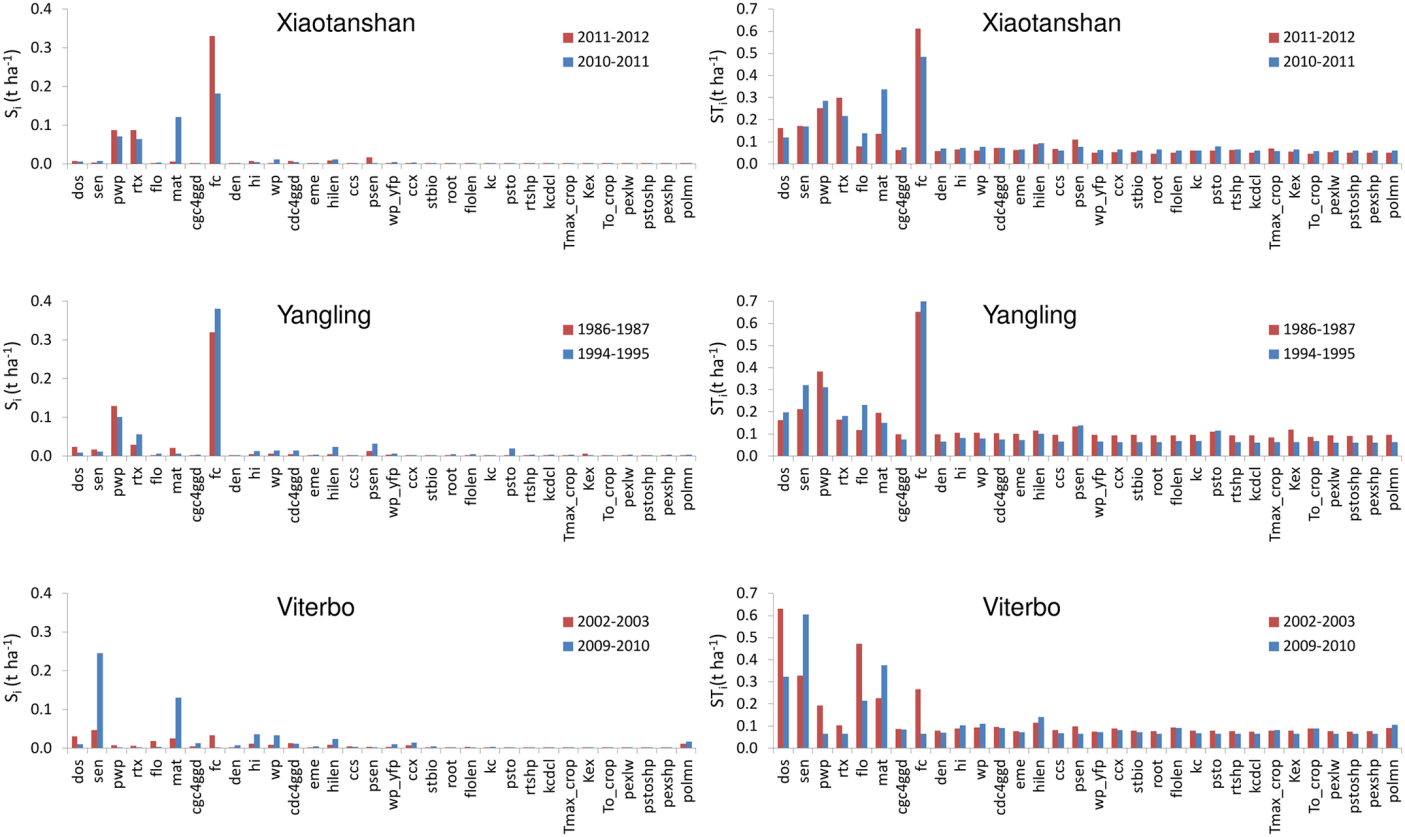
EFAST results for Aquacrop. Main sensitivity index (S_i_) (left panels) and Total Sensitivity index (ST_i_) (right panels), for the three sites, Xiaotanshan (top), Yangling (centre) and Viterbo (bottom), for the two wheat growth seasons examined (wet years bars are blue and dry years red). Abbreviations of parameters and input factors are reported in S1 Table.

Fig 5 shows the two EFAST Sensitivity indices, calculated for Aquacrop for the three locations, for each crop season. The indices represent individual first-order effects, i.e. main sensitivity index (S_i_), and first order plus interactions of higher order with other parameters, i.e. the total sensitivity index (ST_i_) on the variance of model output, i.e. grain yield. Some differences in the degree of sensitivity to what was found using the Morris method are apparent. It should be noted that the Morris sensitivity index μ* includes both first order effects and interactions, so it should be compared to ST_i_ rather than to S_i_. TDCC was computed, to assess the correlation (for common factors) among the rankings of μ* provided by Morris and those of EFAST based on ST_i_, revealing similarities between the results of the two methods. It was found that the correlation between Morris and EFAST results was higher among the dry years for Yangling and Viterbo (TDCC respectively of 0.86 and 0.91), whereas for Xiaotangshan it was higher in the wet year (0.84) than in the dry year (0.56).

EFAST revealed that interactions and second order effects were overall very high for Aquacrop, as shown by the fact that few factors had strong first order effect, revealed by high S_i_ (Fig 5). These included soil water holding properties (field capacity and wilting point, *fc* and *pwp*) especially at Xiaotangshan and Yangling, in which much drier conditions prevailed as compared to Viterbo. The length of the crop cycle (*mat*) and the maximum effective rooting depth (*rtx*) had also a quite important first order effect. The temperature sum to senescence (*sen*) had a relevant first order effect only for Viterbo. Considering the total sensitivity index (ST_i_), three parameters were found to be influential (ST_i_ >0.1) across all sites and years. These were the day of sowing (*dos*) and the phenological parameters indicating the growing degree days from sowing to maturity (*mat*) and to senescence (*sen*). Another important phenological parameter, GDD to flowering (*flo*) was also very influential except for the dry year at Xiaotangshan. Soil field capacity (*fc*) and wilting point (*pwp*) were influential in all scenarios considered except for the wetter year in Viterbo. The maximum effective rooting depth (*rtx*) contributed markedly on the output variance for all sites and years except again for the wet year at Viterbo. These results seem to confirm that even in the years selected as relatively wet for the other two sites, conditions of crop water stress occurred.

Adopting the threshold of the total sensitivity index to identify influential factors (ST_i_ >0.1), considering the maximum values found across all the scenarios, 4 input variables and 13 parameters, of which 5 were conservative, were selected (Table 4).

**Table 4:** List of factor of the Aquacrop model resulting highly influential on the grain yield, according to the main sensitivity index (ST_i_>0.1) from the EFAST analysis, ranked from the most to the least influential.

Name of parameter or input variable	Type	Process affected	Description
fc	Input variable	Soil water stress	Soil Water Content at Field Capacity
dos	Input variable	all	day of sowing
sen	Non conservative parameter	Development of green canopy cover	GDD from sowing to start senescence
flo	Non conservative parameter	Flowering	GDD from sowing to flowering
pwp	Input variable	Soil water stress	Soil Water content at Wilting Point
mat	Non conservative parameter	Development of green canopy cover	Length of the crop cycle
rtx	Input variable	Development of root zone	Maximum effective rooting depth
hilen	Non conservative parameter	Harvest Index	Period of Harvest Index build up during yield formation starting at flowering
psen	Conservative parameter	Soil water stress	Soil water depletion factor for canopy senescence: upper threshold
Kex	Non conservative parameter	Soil water stress	Soil evaporation coefficient for fully wet and non-shaded soil surface
psto	Conservative parameter	Soil water stress	Soil water depletion fraction for stomatal control: upper threshold
wp	Conservative parameter	Crop water productivity	Water productivity normalized for ETo and CO_2_
hi	Non conservative parameter	Harvest Index	Reference Harvest Index
polmn	Conservative parameter	Air temperature stress	Minimum air temperature below which pollination starts to fail
cdc4ggd	Conservative parameter	Development of green canopy cover	CDC for GGD: decrease in canopy cover

**Fig 6:**
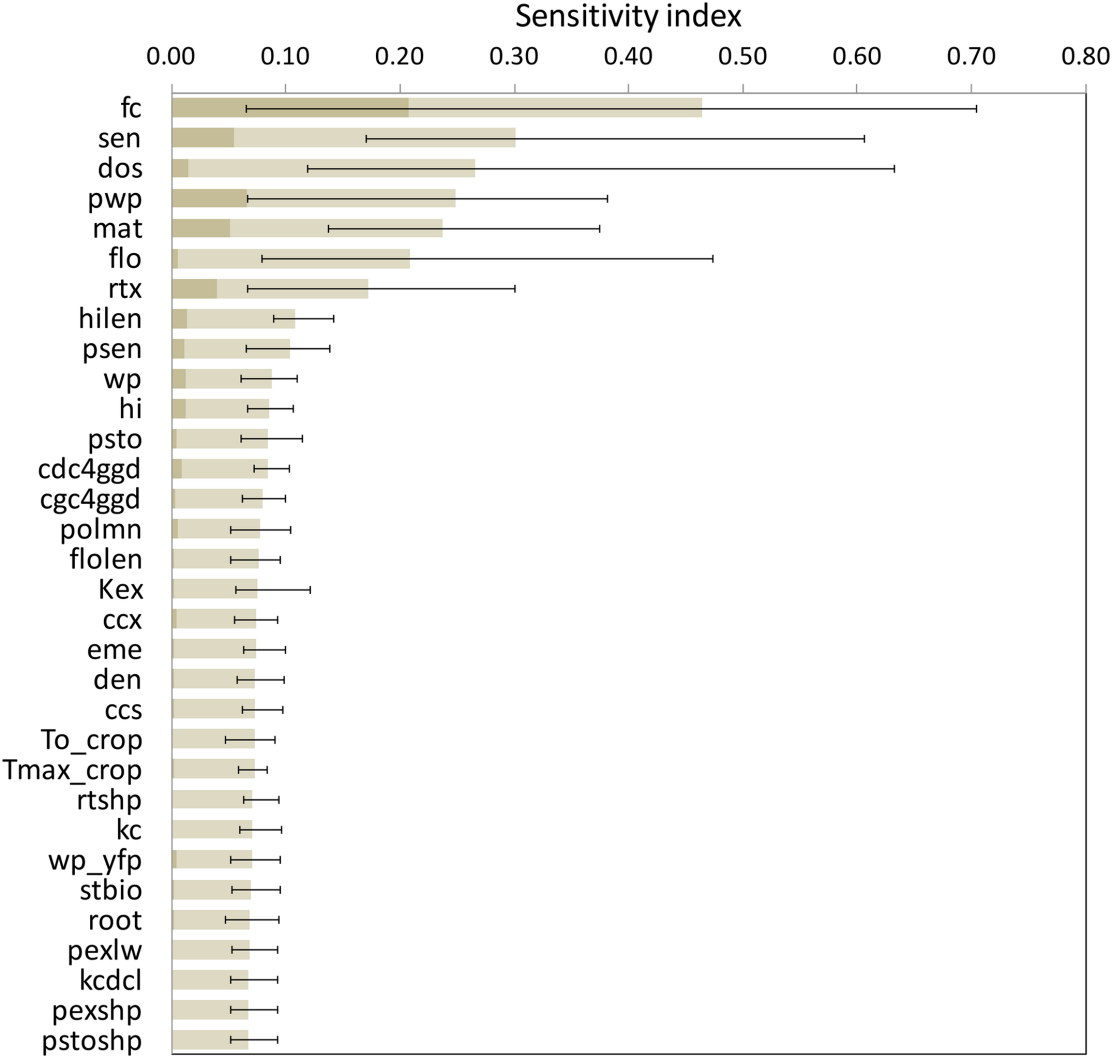
First-order and total sensitivity indices estimated by the EFAST method for the Aquacrop model. The first part of the bars (dark grey) corresponds to the average estimate of the first-order index (S_i_) over all the sites and scenarios, the full bars indicate average estimates of total index (ST_i_), while the lines indicate extreme estimates of total indices.

However it can be observed that additional parameters had a smaller, but still possibly non negligible influence on yield (Fig 6). The correlation (TDCC) among the sensitivity analysis results obtained in the different scenarios for Aquacrop was higher for EFAST, with a TDCC of 0.71, than for Morris (0.47). Contrarily to what observed with the results of Morris, a higher correlation was found among the results of the wet years (0.86) than of the dry years (0.65), but also in this case it was found that Viterbo (TDCC = 0.68) was the site in which the two years were the most different as compared to the other two sites (0.89 and 0.93).

**Fig 7:**
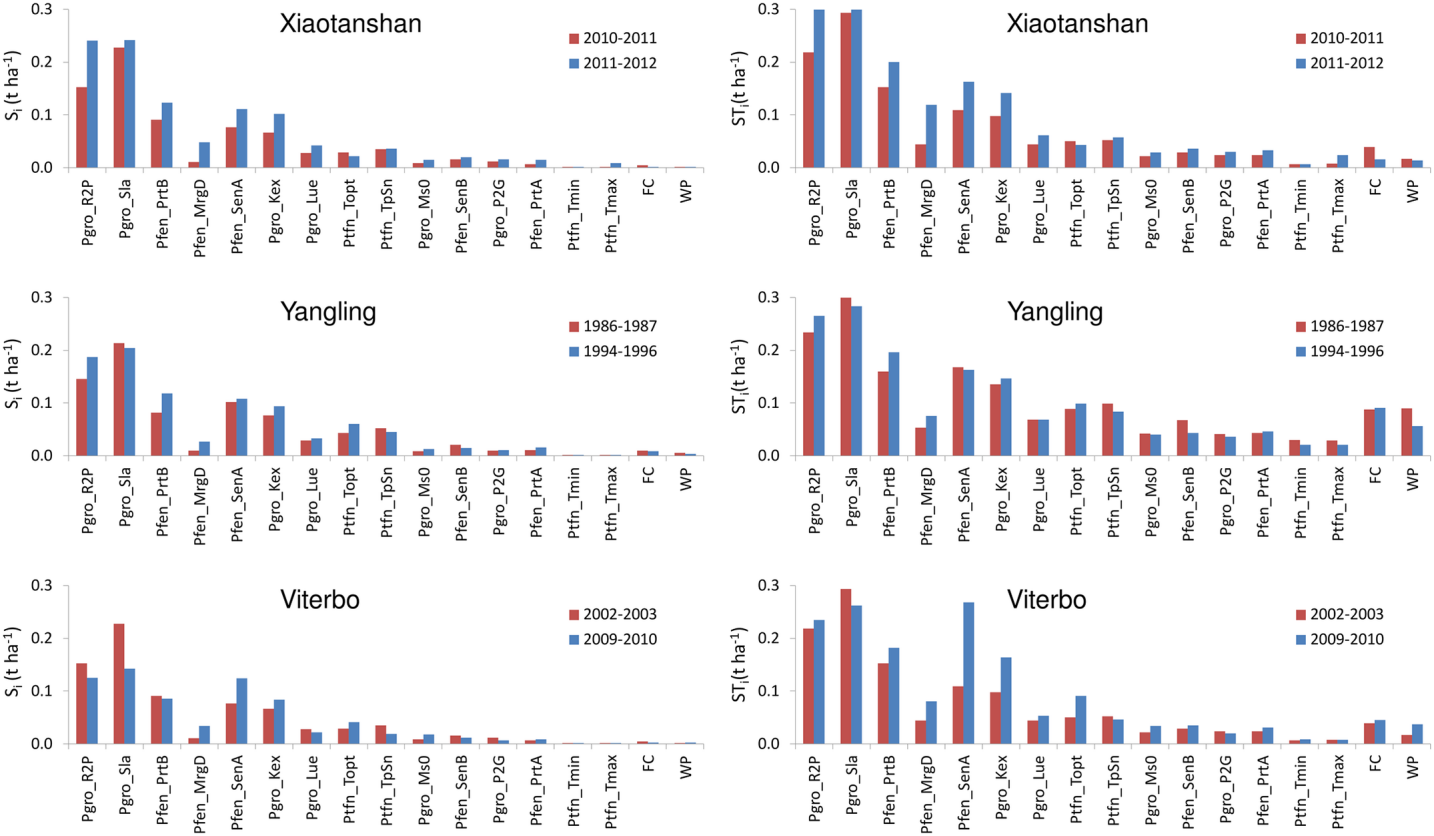
EFAST results for SAFYE. Main sensitivity index (S_i_) (left panels) and total sensitivity index (ST_i_) (right panels), for the three sites, Xiaotanshan (top), Yangling (centre) and Viterbo (bottom), for the two wheat growth seasons examined (wet years bars are blue and dry years red). Abbreviations of parameters and input factors are reported in S2 Table.

The EFAST results for SAFYE (Fig 7) revealed that for this model, first order effects were relatively more important than for Aquacrop. The similarity between the rankings obtained from Morris and EFAST was higher for SAFYE as compared to Aquacrop. For SAFYE the TDCC values between the results of the two methods ranged between 0.83 (Viterbo wet year) to 0.96 (Yangling wet year). Factors showing noticeable EFAST main sensitivity index values concerned especially the climatic conversion factor from solar radiation to PAR (*Pgro_R2P*) and the specific leaf area (*Pgro_Sla*) already shown to be very influential from the Morris method. A high total effect on the output variance, i.e. including also interactions and second order effects, was shown by 4 parameters. In addition to *Pgro_R2P* and *Pgro_Sla*, these included also the parameter regulating the partitioning of biomass to leaves (*Pfen_PrtB*) and the temperature threshold to start senescence (*Pfen_SenA*). The light extinction coefficient in the canopy (*Pgro_Kex*) was also found to be generally influential, except for the dry year in Viterbo and the wet year in Xiaotangshan. The emergence date (*Pfen_MrgD*) was only relevant for the dry year in Xiaotangshan. These different results were nevertheless more correlated among them than those of Aquacrop, yielding in this case a TDCC of 0.93. For SAFYE the correlation among rankings of ST_i_ was roughly similar for wet and dry scenarios, with TDCC values of 0.94 and 0.93 respectively. Also in this case it was found that the results of Viterbo (TDCC = 0.81) were less similar among themselves than those of the two other sites (TDCC of 0.98 and 0.97).

When taking into account the threshold set for considering a factor as influential, applied to the maximum ST_i_ across all climate and site scenarios, only 5 parameters and one input variable were selected (Table 5). The influential factors were the same for both dry and wet scenarios, with the exception of *Pfen_MrgD* which had a strong effect on the output variance (ST_i_>0.1) only in the dry year in Xiaotangshan.

**Table 5:** List of factor of the SAFYE model resulting highly influential on the grain yield, according to the main sensitivity index (ST_i_>0.1) from the EFAST analysis, ranked from the most to the least influential.

Name of parameter or input variable	Type	Process affected	Description
Pgro_Sla	Conservative parameter	Development of green canopy cover	Specific Leaf Area (m^2^ g^-1^)
Pgro_R2P	Conservative parameter	Radiation environment	Climatic efficiency: ratio of incoming photosynthetically active radiation (PAR) to global radiation
Pfen_SenA	Non conservative parameter	Development of green canopy cover	Temperature threshold to start senescence (°C)
Pfen_PrtB	Non conservative parameter	Development of green canopy cover	Partition to leaf function parameter 2 (PLb)
Pgro_Kex	Non conservative parameter	Development of green canopy cover	Light extinction coefficient in canopy
Pfen_MrgD	Input variable	Development of green canopy cover	Day of the year of emergence

**Fig 8:**
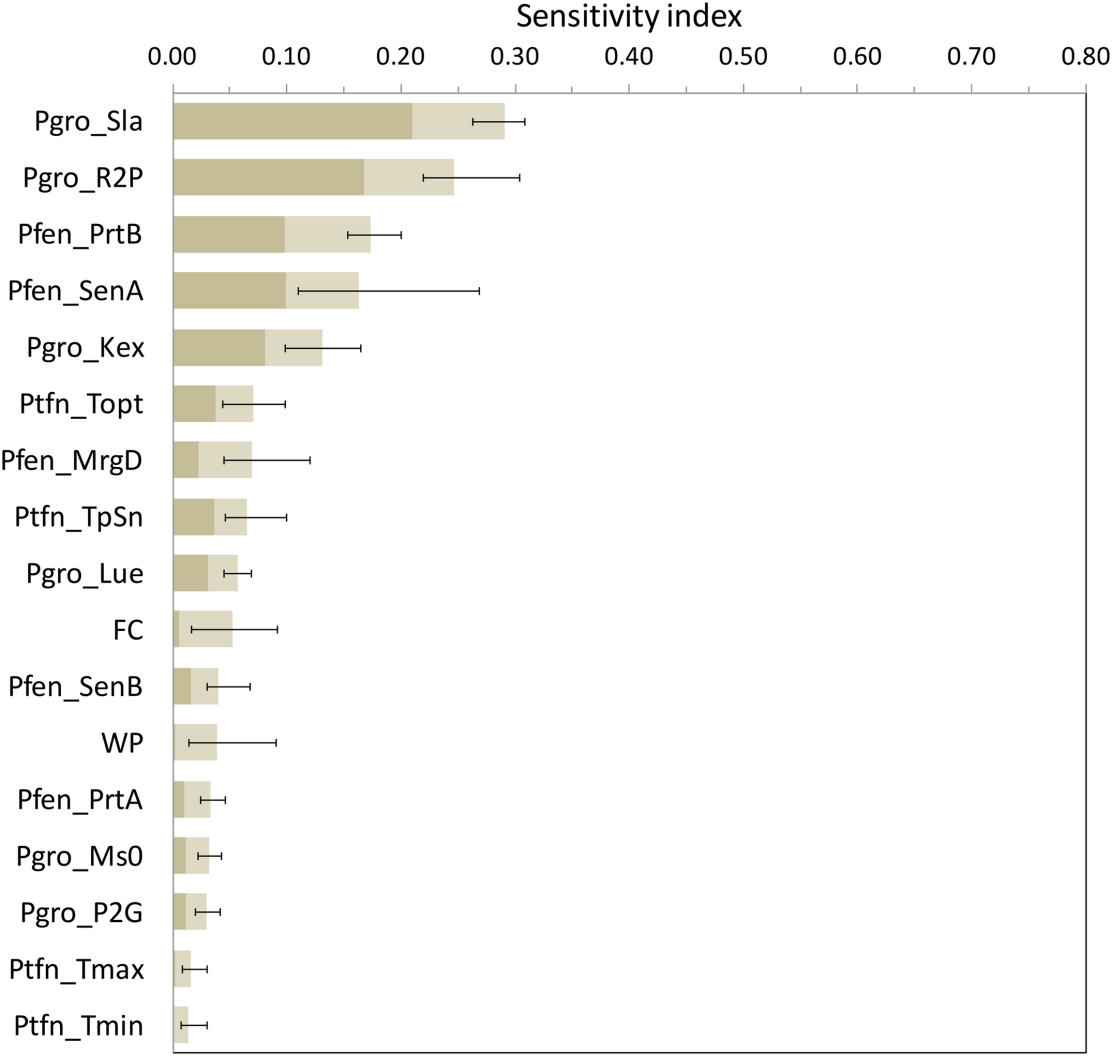
First-order and total sensitivity indices estimated by the EFAST method for the SAFYE model. The first part of the bars (dark grey) corresponds to the average estimate of the first-order index (S_i_) over all the sites and scenarios, the full bars indicate average estimates of total indices (ST_i_), while the lines indicate extreme estimates of total indices.

Other parameters also important were found to be in particular two parameters linked to temperature stress (*Ptfn_Topt* and *Ptfn_TpSn*) and the light use efficency (*Pgro_Lue*) (Fig 8).

### Complexity and plasticity of Aquacrop and SAFYE

The results of the EFAST sensitivity analysis allowed to quantify the complexity and the plasticity of Aquacrop and SAFYE. The complexity of these two crop models was measured using the parameter ratio (R_p_). For Aquacrop R_p_ had a value of 0.28, indicating that 28% of the factors tested (15 out of 54), i.e. including both parameters and input variables, were found to be influential. For SAFYE a lower complexity index R_p_ of 0.19, was found, since 6 factors out of the 31 tested were found to be influential.

The plasticity of the models was calculated by the index L (Eq 4). The results are summarized in Table 6, which shows that all the scenarios employed in this study were characterized by an evapotranspiration deficit, as testified by the SAM values lower than zero. The largest variability in the climatic conditions between the dry and wet years occurred at Viterbo and the lowest at Yangling. In general, the climatic variability across sites was higher among the wet years than among the dry years.

**Table 6:** Synthesis of the assessment of the plasticity of the SAFYE and Aquacrop models. The plasticity index L is calculated according to Eq 4 from the normalized synthetic agrometeorological indicator (SAM) (Eq 5) and its standard deviation σ_SAM_ and the top-down concordance coefficient (TDCC) [5,3].

	SAM	σ_SAM_	TDCC		L	
			SAFYE	Aquacrop	SAFYE	Aquacrop
Site						
Xiaotanshan	-0.17	0.18	0.98	0.89	0.43	0.39
Yangling	-0.34	0.15	0.97	0.93	0.41	0.40
Viterbo	-0.02	0.26	0.81	0.68	0.38	0.32
Year						
wet	-0.04	0.20	0.94	0.86	0.42	0.39
dry	-0.32	0.12	0.93	0.65	0.39	0.27
Total	-0.18	0.21	0.93	0.71	0.42	0.32

The TDCC was always higher for SAFYE than for Aquacrop. This meant that a higher plasticity, indicated by lower L values, was always found for the latter model. The largest differences between the two models in terms of TDCC values, and hence of L, were found between the two years at Viterbo and among the three scenarios of the dry years, where the Aquacrop model demonstrated a much higher plasticity than SAFYE.

## Discussion

The results obtained in this study, performed using a contrasting range of water limited climatic scenarios of winter wheat growing areas, allowed to obtain essential information on the sensitivity and plasticity of two models, SAFYE and Aquacrop, especially required for their increasing application within regional scale studies [7, 13, 22, 24, 56].

In these conditions, there is typically greater uncertainty on parameters and input factors, as compared to field based application of crop models. Therefore it is important to know on which factors data collection and calibration efforts should concentrate. Global sensitivity analysis is considered as the state of the art technique for such purpose [35].

It is well known that the results of sensitivity analysis studies depend on to the "boundary conditions" chosen [57]. In this specific case, these conditions are the climate dataset, actual data from three different sites, and the range of variation of parameters and input variables. The range of variation of these factors was chosen to be the same for all the tests of a given model and, as much as possible, a similar (or the same) range of variation was adopted for analogous factors of the two models. This was done in order to reduce the variability of the boundary conditions of the SA and provide some possible element of comparison between the models. The climatic conditions are therefore the only variables that differentiate the scenarios between the sites. Thus it is possible to distinguish more simply if the parameters are always influential or always negligible, with similar degrees of influence for any scenario, or if their degree of influence varies with the climatic dataset. The climatic data using in this study were chosen to be representative of situations of moderate to strong water deficit, since the main interest was that of providing tools for the assessment of the impact of such conditions on wheat yield, at the regional scale [7]. Thus the results of this study can apply to similar climatic scenarios, which characterize quite a large extent of the wheat growing areas worldwide. The overall value of the σ_SAM_, as reported in Table 6, indicates that the variability among the climatic scenarios used in the present work was very high, being higher for instance than the overall value of the dataset used by [51] which included locations all over Europe (e.g. from Ukraine to Greece) for 10 years, i.e. conferring robustness to our results.

The different climatic scenarios led to some differences in the ranking of the sensitivity of the two models for both GSA techniques, Morris and EFAST. It is normal that the sensitivity to some factors might differ among climatic scenarios, since for example, a parameter that determines the effect of water stress will be important in situations where there is actually water stress, but not where water is non-limiting. This aspect affect the results of all sensitivity analysis studies [57]. Our results, though, revealed clear patterns, allowing to classify groups of parameters as influential or negligible, even in highly contrasting climatic scenarios. We used an objective measure, the top down correlation coefficient (TDCC) to assess the agreement among the listing and ranking of factors assessed across different climates. It was found that the correlations (TDCC values) were always statistically significant (P<0.01), using the methodology to testing its significance reported by Confalonieri et al. [51]. The correlation of the results of the two EFAST rankings of the wet and dry years in a given location ranged between 0.68, for Aquacrop in Viterbo, to 0.98, for SAFYE in Xiaotangshan (Table 6). The correlation among rankings across sites ranged from 0.65 for Aquacrop in the dry years, to 0.94 for SAFYE in the wet years. Overall, it emerged a clear picture, showing that SAFYE had a lower plasticity than Aquacrop, for which the sensitivity analysis results were more affected by the differences in the climatic scenarios.

The impact or the climate scenarios on the sensitivity analysis results, as quantified by the TDCC values, was much higher for Morris than for EFAST, thus the latter was confirmed to be a more robust method [36, 45], providing more stable listings and rankings of the sensitivity index across scenarios. The overall TDCC (across all years and sites) was 0.47 for Aquacrop with Morris and 0.71 for EFAST, for SAFYE it was 0.92 with Morris and 0.93 with EFAST. The agreement between the rankings of the sensitivity indices obtained with the two methods, respectively σ* and ST_i_, for the same set of parameters (i.e. only those in both analyses), was further assessed by using the TDCC across methods for each scenario. It emerged, as also illustrated by comparing Fig 3 with Fig 5 and Fig 4 with Fig 6, that there was an agreement between the rankings of the Morris and EFAST methods. The TDCC varied between a minimum of 0.56 for the dry year at Xiaotangshan to a maximum of 0.96 for SAFYE in the wet year at the Yangling site. On average the agreement between the rankings of the two methods was higher for SAFYE, with a mean TDCC of 0.91, than for Aquacrop which had a mean TDCC of 0.74.

However, the rankings obtained by the Morris method are not always stable. As illustrated by [40], a much higher number of trajectories, than the one used in this study (25), would be required to have more stable results, possibly more than 400, making the computing excessively time consuming. For these reasons, the Morris method, as reported by [40] and [45], can only be considered useful for screening out negligible factors, whereas its sensitivity rankings can only be considered as qualitative and not quantitative.

For the Aquacrop model, the set of negligible parameters, identified in this study by the Morris method, coincides almost completely with the one identified by Vanyutrecht et al. [40]. The most notable differences concern the soil curve number (*CN*) and the shape factor for water stress coefficient inducing early senescence (*psenshp*), which were classified as negligible in the present study, whereas they were found to be very influential by [40]. The *CN* is used by Aquacrop for the estimation of the amount of rainfall lost by surface runoff [41], so it is understandable to have had a limited impact, in the water limited situations of the scenarios used in the present study. In Aquacrop the shape of the water stress coefficient curve can be selected as linear or concave by changing *psenshp*. As observed by [41], shape parameters are highly intertwined with the critical level parameters of the stress coefficients. However, the former have no physical meaning, whereas the latter have physical sense and can more easily be calibrated. In our case, the parameter regulating the specific stress coefficient, i.e. soil water depletion threshold for triggering early canopy senescence (*psen*), was found to be very influential, similarly to what found by [41].

The set of Aquacrop negligible parameters listed in Table 2, in all the scenarios tested in the present study, have a limited impact on the variation of the output, so it is possible to exclude them from calibration or assimilation methods, or sensitivity analysis for climatic scenarios similar to those analyzed in this study.

For SAFYE, the results of the Morris method indicated very clearly that all the parameters and input factors describing the water balance and related stress function, introduced into the original SAFY model (to turn it into SAFYE), were negligible, except for soil moisture at field capacity and wilting point (Table 3). The latter two parameters, however, were not in the top ranking of the EFAST analysis (Fig 8). The implication of this finding are that, if the purpose of the study is only the assessment of wheat yield, even in conditions of severe water stress the original SAFY model is appropriate. Indeed it would even be a better choice than SAFYE, since it relies on a more limited number of parameters and input factors. If, on the contrary, information is sought on the incidence and patterns of water stress, separately from other stress factors (e.g. nitrogen), SAFYE is to be preferred, though a simplification of its water stress components is suggested.

In agreement with the literature [40, 57], the results of this study confirm that the application of the Morris method is efficient for the identification of the negligible parameters, but less so for the classification of the parameters' sensitivity. Looking at the rankings of the parameters with respect to the Morris μ* (Figs 3 and 4) it is possible to note that the weather conditions had a more significant effect than for EFAST (Figs 5 and 6), as confirmed by the higher TDCC of the former tests. The ranking of parameters sensitivity is expected to be more reliable for EFAST, as previously mentioned, since the TDCC values across sites and years were much higher than for Morris. For both models it was possible to establish a set of influential parameters for all considered scenarios (Tables 4 and 5). EFAST allowed also to discriminate the effect of first order effects of single factors from second order effects and interactions on the output variance, revealing interesting difference among the models.

For Aquacrop it was found that very few factors had a strong first order effect, so that their impact on the grain yield was mainly due to second order effects and interactions with other parameters (Fig 6), revealing a rather complex model behavior. A higher predominance of first order effects was found by [40], however with different crop species and with more diversified climate scenarios from those we used, highlighting more clearly first order effects. For SAFYE first order effects had a stronger impact on yield variance (Fig 8).

The ranking of the total sensitivity index for Aquacrop (Fig 6) was different from the one obtained by [40], since as mentioned earlier, this reflects the relative importance of specific factors addressing processes occurring in the different climatic scenarios. In our case, the factors that had the highest impact on yield variance were mainly those related to water stress as well as phenological parameters regulating the development of the green canopy cover.

For SAFYE, the most influential factors were those related to the development of leaf area and leaf biomass. This confirms that, in this model, the impact of water stress on crop yield is modeled indirectly from its impact on LAI, rather than explicitly through the water stress parameters introduced into the original SAFY model.

For both models used, Aquacrop and SAFY, the parameters describing the phenological cycle of the crop played a decisive influence on the estimation of crop yield, being influential for both models. The coincidence between phenological stages and periods of the year with certain temperature and rainfall is among the causes that most affect the variation in model output, especially for Aquacrop.

From the literature [57] it is known that choices made on the ranges of parameters and input variables influence the ranking of the sensitivity indices. For both Aquacrop and SAFYE these ranges were chosen after a careful inspection of the literature (S1 and S2 Tables), within physically plausible ranges, expected to be found when the models are applied to regional scale studies. In this case, it is important to include also input variables in the SA, in addition to model parameters, because it is interesting to know, especially in a regional scale model application, what is their impact on the output. Many authors have made it clear that SA should not be necessarily strictly limited to parameters, but can also include input variables (see e.g. Wallach et al. [14] or Saltelli et al. [35]).

For Aquacrop we tested in the SA also conservative parameters [41], that are supposed to be fixed for a given species, but in reality, the calibration results of different studies on wheat [27–30, 33–34] suggest that even for these parameters a limited range of variation occurs. Thus we adopted, for these parameters, ranges resulting from these calibration studies (with a maximum of ±33%), including them in the SA, as also done by Vanuytrecht et al. [40].

As expected, the SA carried out for Aquacrop was more complex than that done for SAFYE. Aquacrop describes more accurately than SAFYE the crop growth processes, using a higher number of equations and input variables. Therefore the SA performed for the former model had a much higher computational cost than for the latter. Using a computer with a Intel(R) Core(TM) i7-4770 CPU processor at 3.40 GHz, and 16 GB of RAM, the computation time for applying EFAST to Aquacrop was in the order of hours, while for SAFYE in the order of minutes. SA is a step required for any application of assimilation or calibration, thus the computational cost could be a factor that affects the choice of the model to use.

Both complexity and plasticity were found to be higher for Aquacrop than for SAFYE (Table 6). Confalonieri et al. [50], in a comparison of three crop models, found that the model having the highest complexity (WOFOST) was the one with the highest plasticity, but also the lowest robustness. They considered that a high value of plasticity was a positive aspect for a crop model, seeming to reflect the phenotypic plasticity typical of crops. However, in the context of data assimilation, or of applications of crop models at a regional scale, a high plasticity would be a nuisance, since it might not allow to clearly identify for which parameters the effort to reduce their uncertainty needs to be focused. As Ben Touhami et al. [58] have pointed out, a high plasticity complicates the interpretation of the SA results and may require checking how and to what extent the ranking of influential parameters changes with changing conditions. If a high plasticity depends on the listing of parameters, rather than on their ranking, it would limit the generalization capability of a crop model, strengthening the need for site-specific calibrations and sensitivity analyses. In the present work, the plasticity for Aquacrop (L = 0.32) was higher than that found by [50] for the model having the highest plasticity among those tested, i.e. WOFOST (L = 0.37). The high plasticity was determined by differences in the listings (and not only ranking) of the most influential factors for Aquacrop among the scenarios tested. Instead, SAFYE had a lower plasticity, with an L of 0.42, i.e. intermediate between that of the models Cropsyst (L = 0.40) and WARM (L = 0.44) and the listings were the same in all scenarios, with the exception of parameter Pgro_Kex which was not influential in 2 scenarios only.

## Conclusions

The results reported in this study provide key elements to the knowledge on the behavior, in water limited climatic scenarios, of two models, Aquacrop and SAFY, which have an increasing user community and are extremely interesting in the context of regional scale studies, and particularly for remote sensing data assimilation [7, 13, 22, 24, 56]. For these applications, higher uncertainties exist on the values of parameters and input factors needed to run these models, as compared to more usual field based applications of crop models. In the case of large scale applications, it is very useful to know, not only the sensitivity of the targeted model outputs, grain yield in our case, to the parameters and input variables, but also model complexity and plasticity. Sensitivity analysis results provides guidance on the data gathering and calibration efforts, but the knowledge of complexity and plasticity provides elements for the choice of the most suitable model, based on the information available and on the processes that need to be described.

In our case, it was apparent that Aquacrop, despite being simpler than most other crop models, is more complex than SAFYE and it has also a higher plasticity. In a regional scale application of Aquacrop, more attention needs to be paid to its calibration than for SAFYE, which has a smaller number of influential factors. Furthermore, given the higher plasticity of Aquacrop, it would be necessary, more than for SAFYE, to carry out a preliminary sensitivity analysis, in case the scenario in which it would be used differs markedly from those of sensitivity studies already done, such as the present work or [40]. The present study employed a diversified range of climatic scenarios, characterized by moderate to severe water stress, so the results are valid for similar situations which are very frequent in wheat growing areas.

SAFYE is less complex and has a lower plasticity than Aquacrop. It was found that the parameters that were added to the original SAFY model, to include water balance and crop water stress processes, were mostly negligible, with the notable exception of soil moisture at field capacity and wilting point. This suggests that a simplification of the description of these processes in SAFYE should be made. The limited number of parameters and the lower plasticity, makes this model more suitable than Aquacrop to large scale applications, whenever limited information on the input variables is available and an estimate of crop yield is the only information sought. This model, though, lacks the ability of providing insight on the processes that contributed to yield reduction, that would only be made clear by models incorporating the description of influence of the different abiotic (or even biotic) stress factors on crop growth.

## Supporting information

S1 Fig Simulated and measured above ground biomass, grain yield and LAI for the Xiaotangshan winter wheat dataset [30] with different sowing dates in the years 2008–2011. Lines are data simulated with the SAFYE model, points are field measurements.
(PDF)

S1 Table Parameters and input variables of the Aquacrop model considered in the sensitivity analysis, with their minimum and maximum values and the range of variation employed and the references used for setting these values. Conservative and non-conservative parameters and input variables are indicated.
(XLSX)

S2 Table Parameters and input variables of the SAFYE model considered in the sensitivity analysis, with their minimum and maximum values and the range of variation employed and the references used for setting these values. Conservative and non-conservative parameters and input variables are indicated.
(XLSX)
