# Supplementary material for: Sensitivity analysis of the Aquacrop and SAFYE crop models for the assessment of water limited winter wheat yield in regional scale applications
Source: PLoS One. 2017 Nov 6;12(11):e0187485. doi: 10.1371/journal.pone.0187485 (PMC5673191; doi:10.1371/journal.pone.0187485)

## Sowing date: 28 Sept. 2008

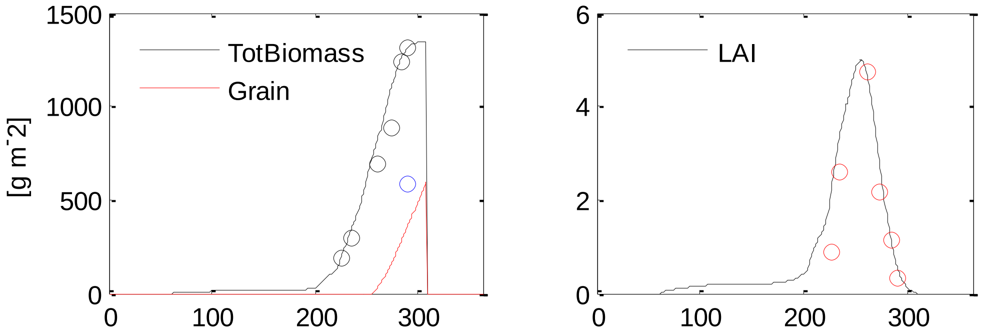


## Sowing date: 07 Oct. 2008

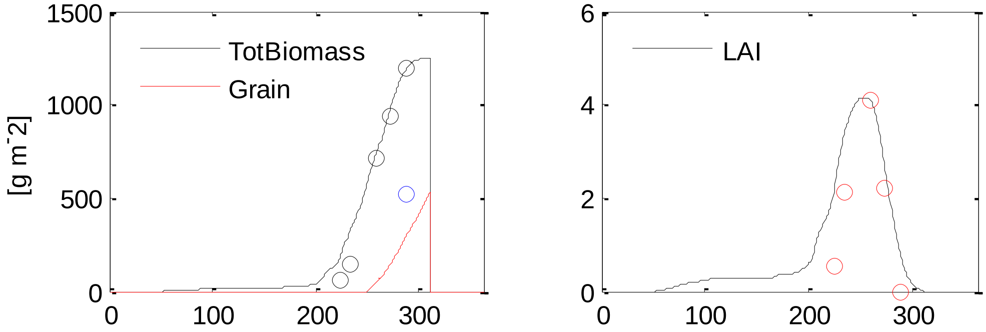


## Sowing date: 20 Oct. 2008

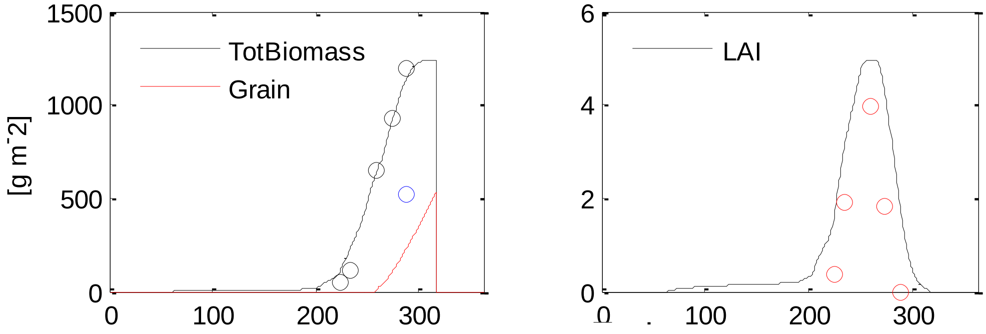

Sowing date: 25 Sept. 2009

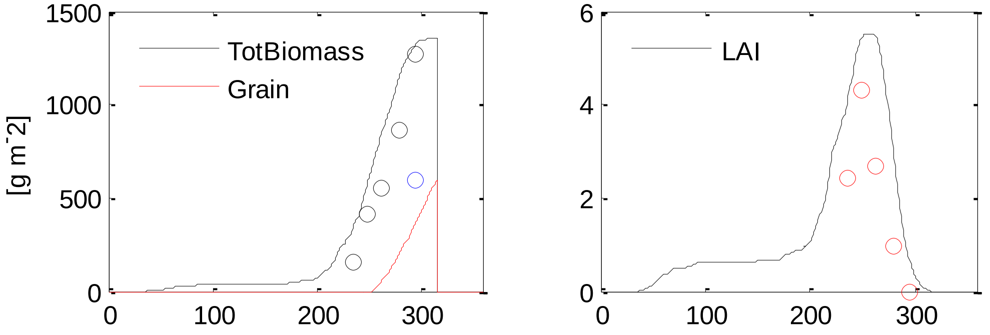


Sowing date: 05 Oct. 2009

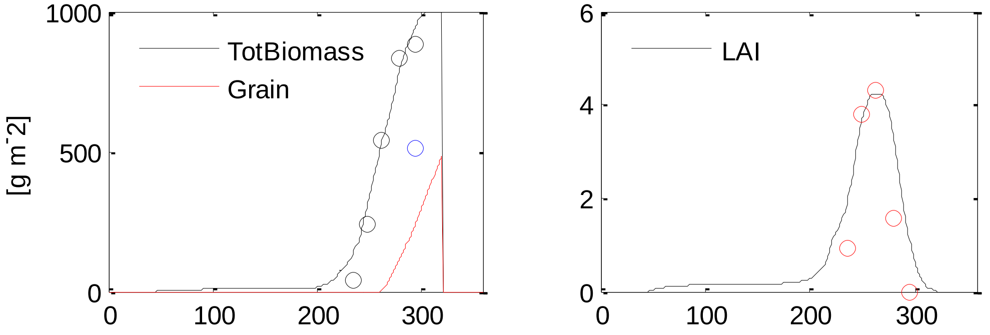


Sowing date: 15 Oct. 2009

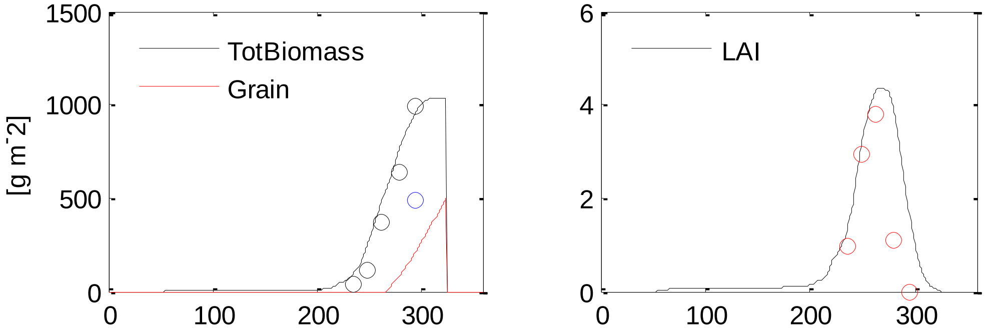

## Sowing date: 05 Oct. 2010

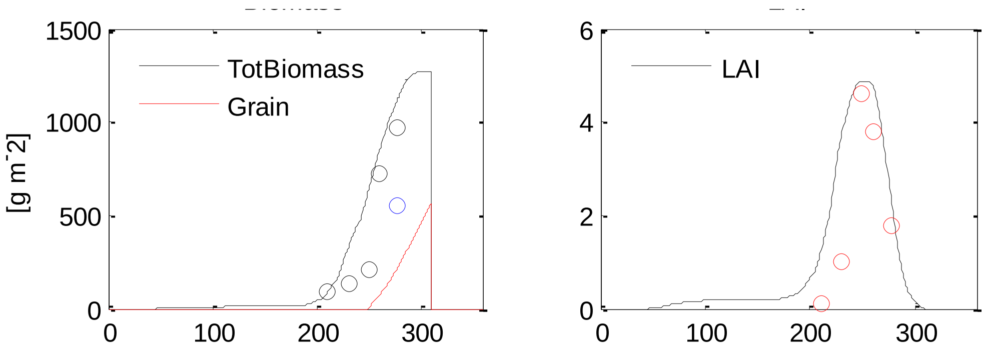


## Sowing date: 15 Oct. 2010

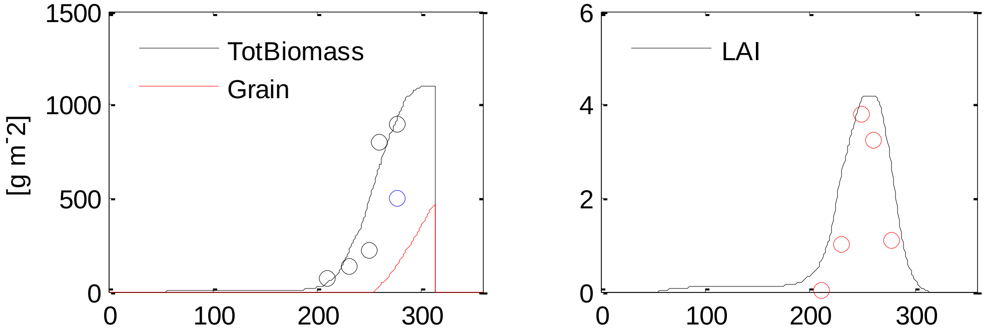


## Sowing date: 25 Sept. 2010

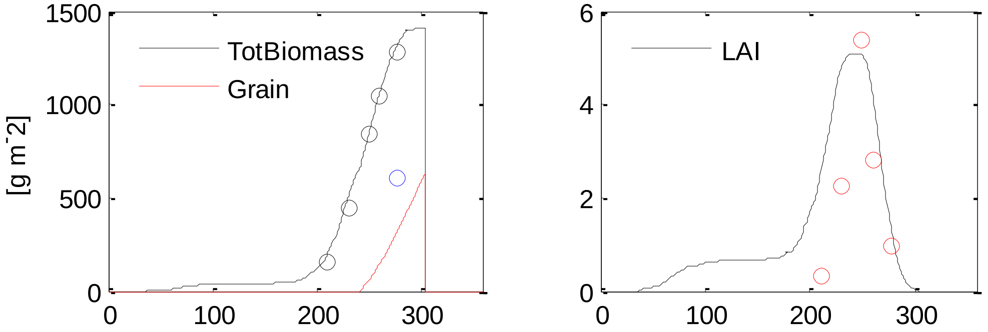


## Sowing date: 25 Sept. 2011

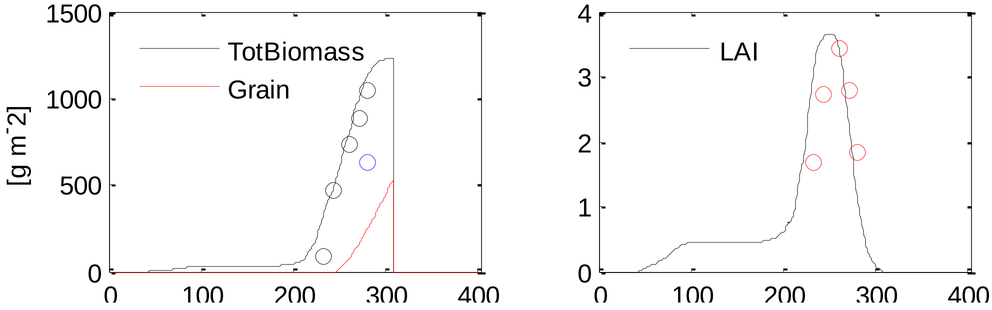

Supplement: S1 Fig — Lines are data simulated with the SAFYE model, points are field measurements. (PDF) [file pone.0187485.s001.pdf]
